# Joint disc and cup segmentation based on recurrent fully convolutional network

**DOI:** 10.1371/journal.pone.0238983

**Published:** 2020-09-21

**Authors:** Jing Gao, Yun Jiang, Hai Zhang, Falin Wang

**Affiliations:** College of Computer Science and Engineering, Northwest Normal University, Lanzhou, Gansu, P.R.China; Ulm University, GERMANY

## Abstract

The optic disc(OD) and the optic cup(OC) segmentation is an key step in fundus medical image analysis. Previously, FCN-based methods have been proposed for medical image segmentation tasks. However, the consecutive convolution and pooling operations usually hinder dense prediction tasks which require detailed spatial information, such as image segmentation. In this paper, we propose a network called Recurrent Fully Convolution Network(RFC-Net) for automatic joint segmentation of the OD and the OC, which can captures more high-level information and subtle edge information. The RFC-Net can minimize the loss of spatial information. It is mainly composed of multi-scale input layer, recurrent fully convolutional network, multiple output layer and polar transformation. In RFC-Net, the multi-scale input layer constructs an image pyramid. We propose four recurrent units, which are respectively applied to RFC-Net. Recurrent convolution layer effectively ensures feature representation for OD and OC segmentation tasks through feature accumulation. For each multiple output image, the multiple output cross entropy loss function is applied. To better balance the cup ratio of the segmented image, the polar transformation is used to transform the fundus image from the cartesian coordinate system to the polar coordinate system. We evaluate the effectiveness and generalization of the proposed method on the DRISHTI-GS1 dataset. Compared with the original FCN method and other state-of-the-art methods, the proposed method achieves better segmentation performance.

## 1 Introduction

**Fig 1:**
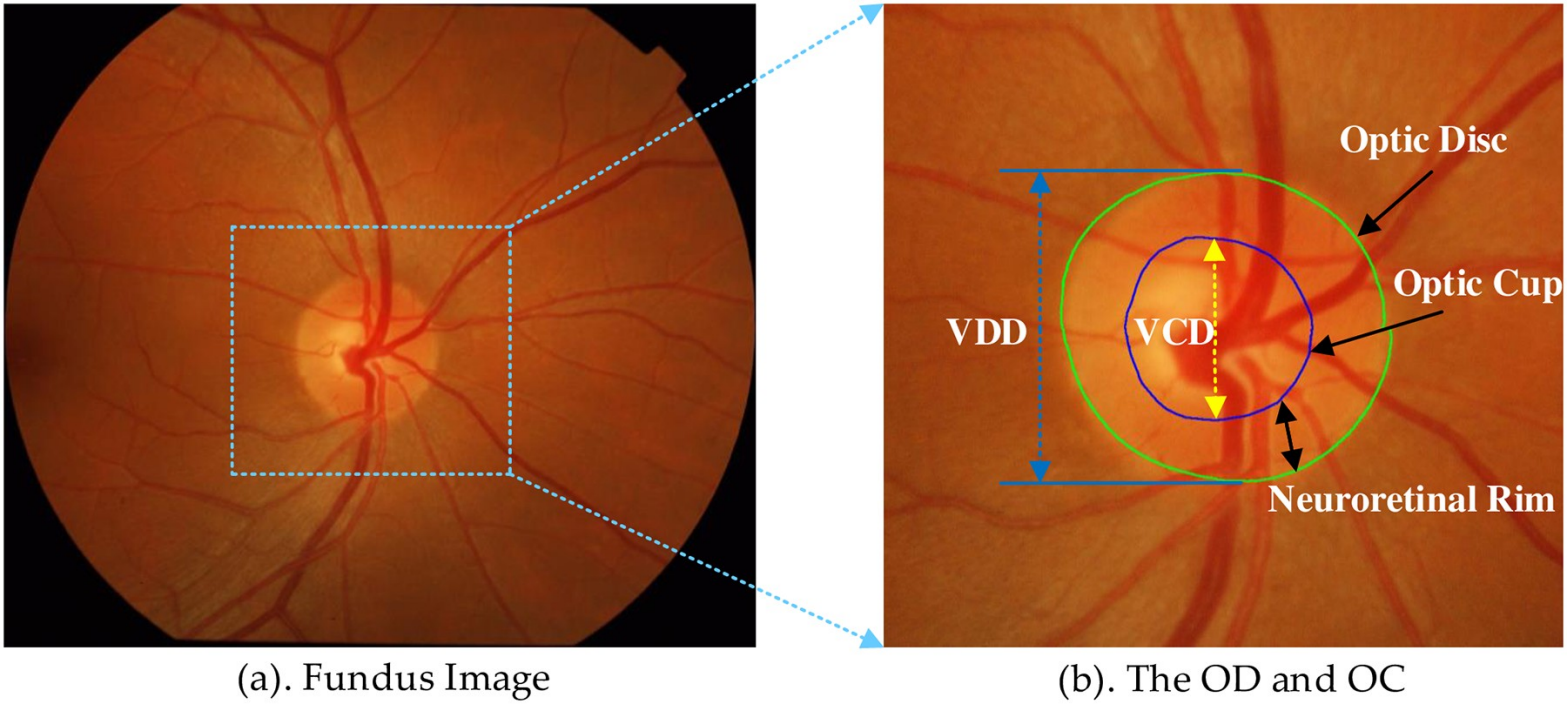
Introduction of OD and OC area. (a) shows the structure of the optic nerve head. (b) shows the optic disc and cup structure.

The optic disc(OD) and the optic cup(OC) segmentation is often an indispensable work in medical image analysis [1]. However, the division of the OD and the OC is a very time-consuming task that is currently only performed by professionals. Therefore, the use of computers to automatically segment the OD and the OC is attractive because the computer is more objective and faster than the human segmentation. In the retinal fundus image, it is very necessary to use the deep learning method to automatically segment OD and OC, which is regarded as one of the most fundamental tasks in this field [2]. It helps to quantify clinical measures about the retinal related diseases, and provides a basis for accurate diagnosis by doctors [3]. For example, OD and OC segmentation plays a key role in the calculation of vertical cup-to-disk ratio (CDR)[3]. In two-dimensional color fundus image, the OD can be divided into two regions: The peripheral region which is the edge of the nerve retina and the OC exhibiting as a pit in centre [4], as shown in Fig 1.

The remaining of this paper is organized as follows: In Section 2, we introduce related work. Section 3 introduces the theory and structure of RFC-Net network. Section 4 gives our experiment result, including the introduction to the Drishti-GS1 dataset, and the experiment settings. Section 5 is the discussion part. We did an ablation experiment between modules. Finally, we make our conclusion in Section 6.

By studying the relevant theories and methods of deep learning for assisted diagnosis of retinal images, and using the powerful feature learning capabilities of deep convolutional neural networks to segment the diseased tissue in retinal images, many problems in assisted diagnosis of the retina can be effectively solved. Efficient and stable automatic retinal image segmentation technology can greatly liberate medical resources and reduce the workload of medical personnel, and at the same time provide medical personnel with valuable medical image references.

The challenge of joint OD and OC segmentation is how to learn an efficient segmentation model with good performance. This paper focuses on the research to improve the accuracy of OD and OC segmentation models, including research on reducing the amount of model parameters, improving the receptive field of the model, and understanding the context. We propose a model based on recurrent fully convolutional network, named RFC-Net. This model extends FCN by adding simple and effective recurrent convolution blocks to optimize the segmentation results. First, we consider the RFC-Net architecture as an encoder-decoder architecture. The work of the encoder is to compress the feature map, and the original feature map quality is reduced. The work of the decoder is to decompress the feature map, using a file with a small amount of information but containing all the key information to restore the original feature map. Therefore, the RFC-Net can spontaneously summarize the essence of the original feature map and improve the resolution of the feature map. The recurrent block increases the network depth while keeping the number of adjustable parameters unchanged through weight sharing. This is consistent with the purpose of the current CNN architecture [5–7]: Using relatively few parameters for more in-depth research. At the same time, we further explored the spatial pyramid model and applied it to RFC-Net. The spatial pyramid model not only extracts multi-scale context information from objects, but also does not require additional learning weights. For each multiple output image, the multiple output cross entropy loss function is applied. To better balance the cup ratio of the segmented image, the polar transformation is used to further improve the segmentation performance.

In summary, there are five contributions in our paper:

1. Recurrent Fully Convolution Network (RFC-Net), for automatic joint segmentation of optic disc and cup was proposed.
2. Four new recurrent units are introduced for the OD and OC segmentation, which are respectively applied to RFC-Net. Different units are generated and compared for comparative analysis. *StackRecurrentUnits* gets the best results.
3. With help of multi-scale input and multiple output, the segmentation performance is effectively improved. The multi-label cross entropy loss function is applied to the image by each multiple output.
4. Because the proportion of the OC of the segmented image is not balanced, the polar transformation is used to transform the fundus image from the cartesian coordinate system to the polar coordinate system. With help of polar transformation, the segmentation performance is improved.
5. Compared with the existing methods, the proposed method achieves better segmentation performance. In the segmentation effect of OD and OC, the F1 are 0.9787 and 0.9058, respectively. The BLE are 3.96 pixels and 15.40 pixels, respectively.

## 2 Related works

At present, the segmentation methods of the OD and OC are mainly divided into the following categories: Shape-based and template matching methods [8–10], models based on deformable and active contours [11–13], and the recent deep learning methods [6, 14–26]. We briefly outline the existing methods below.

### Shape-based and template matching methods

Since the shape of the OD area in the retinal image can be seen as an ellipse, it is brighter than other surrounding areas. These methods try to use elliptic curves to fit the OD area [8–10]. In [8], ellipse fitting and wavelet transform are used to realize automatic OD positioning and contour detection. First, Daubechies wavelet transform is used to approximate the OD area, and then the intensity-based template is used to obtain an abstract representation of OD. In [9, 10], the OD is simulated as a circular or elliptical object, and the transformed morphology and edge detection technology are used to approximate the OD contour.

### Deformable-model and active contours based methods

This kind of method transforms the problem of segmenting images into the problem of solving the minimum value of the energy functional by constructing the energy functional. In [11], the author improved and expanded the original method in two aspects, which made the contour deformed to the position with the minimum energy, and self-clustered into the edge point set and the uncertain point set. By updating the combination of local and global information, the model becomes more robust to vascular occlusion, noise, ambiguous edges and fuzzy contour shapes. The study in [12] applied the fast mixed level set model combining the regional and local gradient information to the segmentation of the OD boundary by initializing the detected OD center and the estimated OD radius. The study in [13] proposed an active contour model based on implicit regions, which combines image information from multiple image channels at target region points to resist changes in and around the OD region.

### Models based on deep learning methods

Deep learning methods [6, 14–23] segment OD and OC by training a large number of data samples to automatically extract features. In [15], OD and OC segmentation using superpixel classification for glaucoma screening is proposed. In [16], an entropy-based sampling technique is introduced to advance the convolution filter to segment the OD and OC from the fundus image. In [6], a network U-Net which relies on the use of data augmentation is proposed, which could use the available annotated samples more efficiently. In [17], a general method based on deep learning for automatic OD and OC segmentation, namely U-Net convolutional neural network, is proposed, which outperforms traditional convolutional networks in terms of the prediction time. In [18], by modeling the depth drop between the OD and OC, a method for jointly segmenting the OD and OC is proposed, which can be used for large-scale screening of glaucoma eye. In [20], a special image segmentation cascade network, Stack-U-Net, is proposed. The Stack-U-Net takes the U-Net networks as building blocks, and it is based on the idea of the iterative refinement. Compared with a single U-Net and the state-of-the-art methods for the investigated tasks, it acheives excellent segmentation performance, without increasing the size of datasets. Later, Fu et al. explores a new M-net structure to joint segment the OD and OC [21]. The DENet structure proposes a collection of four independent neural network flows [22]. In [14], the author proposed a multi-label deep convolutional network model GL-Net combined with a generative adversarial network to segment OD and OC. It reduces the downsampling factor and effectively alleviates the loss of excessive feature information. In [23], the CE-Net was proposed as a context encoder network that not only captures more advanced information, but also preserves spatial information. These recent deep learning methods have performed well and successfully promoted the study of OD and OC segmentation of fundus images from the perspective of deep learning. In [19], the author uses RACE-Net based on a recurrent neural network to simulate a variable model of generalized level sets that evolve at constant and average curvature speeds. It can clearly simulate the high-level dependence between points on the boundary of an object, maintaining its overall shape, smoothness, or homogeneity of the area inside and outside the boundary. Some work [24–26] use the recurrent convolutional network to segment the fundus retinal vessels, multi-slice MRI cardiac and video better capturing local features and enriching context dependencies. The recurrent convolutional network can establish a connection between the first layer and each of the other layers. Deformable-model and active contours based methods.

In summary, the shape-based and template matching methods are more representative in the early stage. However, such methods may collect images with different colors, uneven intensities, and the presence of focus areas, infiltrations, and blood vessels in the OD area, which make these segmentation methods less robust. Deformable-model and active contours based methods are more sensitive to local minimum states, so the global minimum may not be achieved due to noise and focus. In the process of energy minimization, small features are ignored and the convergence strategy has a greater impact on accuracy. The deep convolutional neural network can automatically learn the correlation between the features in the fundus image and is relatively less affected by the lesion. However, for the existing OD and OC segmentation methods based on deep learning, due to the fixed number of network layers, downsampling is generally used to improve the receptive field. When OD and OC are jointly segmented, the OC area on the label map is relatively small. Too large a downsampling factor will cause loss of OC edge information. For a relatively large OD region, the receptive fields of these methods are not large enough, they cannot fully understand the global information, and cannot accurately identify some large segmented regions. Therefore, in order to capture the rich context in the image, we propose a recurrent fully convolutional network model RFC-Net. The concept of recurrent is added to it, and four types of recurrent units are carefully designed to capture more local characteristics and enrich context dependencies. The recurrent convolutional network helps to train the deep architecture. It can expand the receptive field of the model while maintaining the feature relevance, thereby making up for the shortcomings of FCN.

## 3 Recurrent Fully Convolution Network(RFC-Net)

In this section, we first introduce the overall framework of our network and then introduce different modules in the RFC-Net. Finally we describe how to best combine them together for further refine the network.

### 3.1 Overview

Inspired by the recurrent convolutional network [27] and the FCN [5], we propose a deep learning network for segmentation tasks. The deep FCN called RFC-Net is constructed, which solves the joint segmentation problem between OD and OC. Compared the basic FCN with RFC-Net, RFC-Net mainly has the following improvements: (1) Adding polar transformation, (2) adding multi-scale input modules, (3) four recurrent units are proposed and applied to RFC-Net, and adding skip connections in RFC-Net, (4) using multiple output fusion to obtain final segmentation results. In this section, we outline the principles and advantages of the used method.

**Fig 2:**
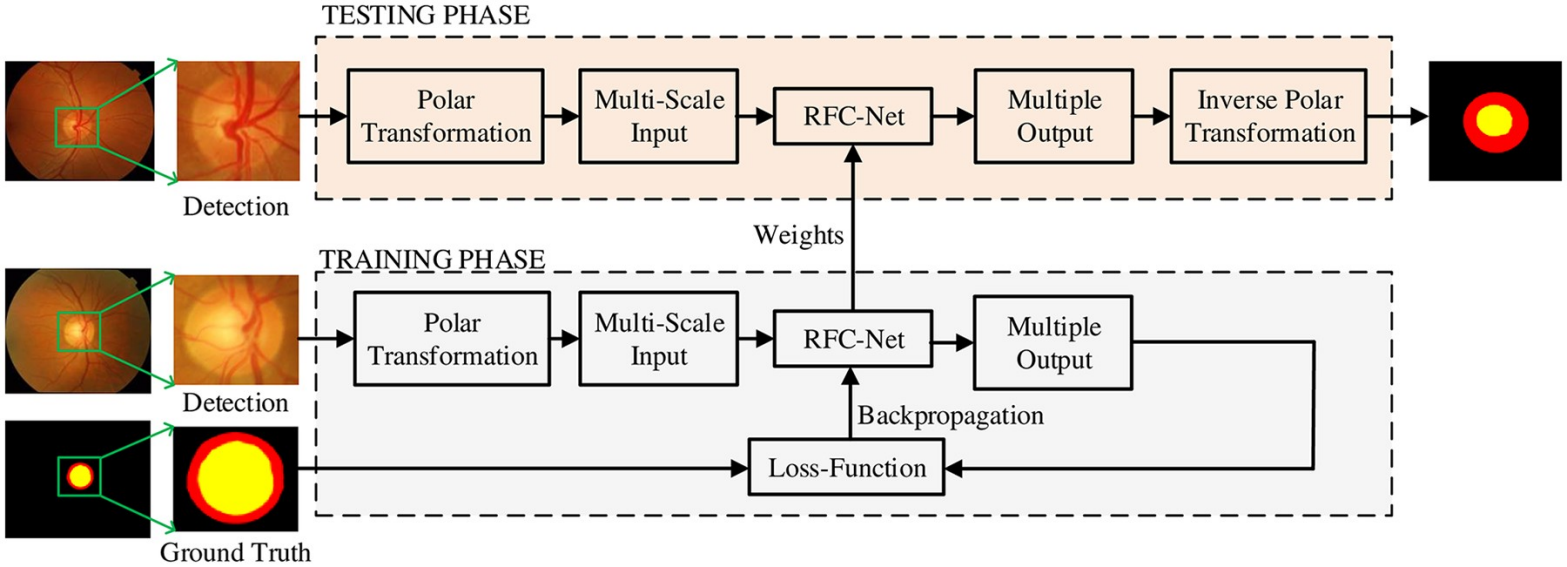
The training and testing process.

Our training and testing process is shown in Fig 2. First we use the existing advanced automatic disc detection method YOLOv2 [28] to locate the disc center, and adopt a network framework based on target detection so that the position of the disc can be identified at the same time without prior selection of the region of interest. Perform preprocessing is performed on the detected images. Next, by inputting a fundus retina image into the polar transformation block, the polar transformation block converts the fundus retina image from the cartesian coordinates to the polar coordinate system, and outputs the fundus retina image in the polar coordinate system. Polar transformation further improves the segmentation performance of OD and OC. We down-sample the images in the network, then create a multi-scale input in the encoder path, input feature images in a multi-scale manner, and encode multi-size context information. We use RFC-Net as the main network structure to learn rich hierarchical representations. The output layer is used as an early classifier for generating accompanying local prediction maps of different scale layers. The number of channels of the multiple output image is 32, 64, 128, 256. We scale the multiple output feature map to 3 channels through 3 × 3 convolution, and the feature map is finally classified into three categories: 0 corresponds to background, 1 corresponds to OD, and 2 corresponds to OC. A cross entropy loss function is applied to each multiple output layer image, and the output map for each scale is supervised to output better results. Finally, the segmented image is restored to the cartesian coordinate system through the inverse polar transformation and the final segmented image is output.

**Fig 3:**
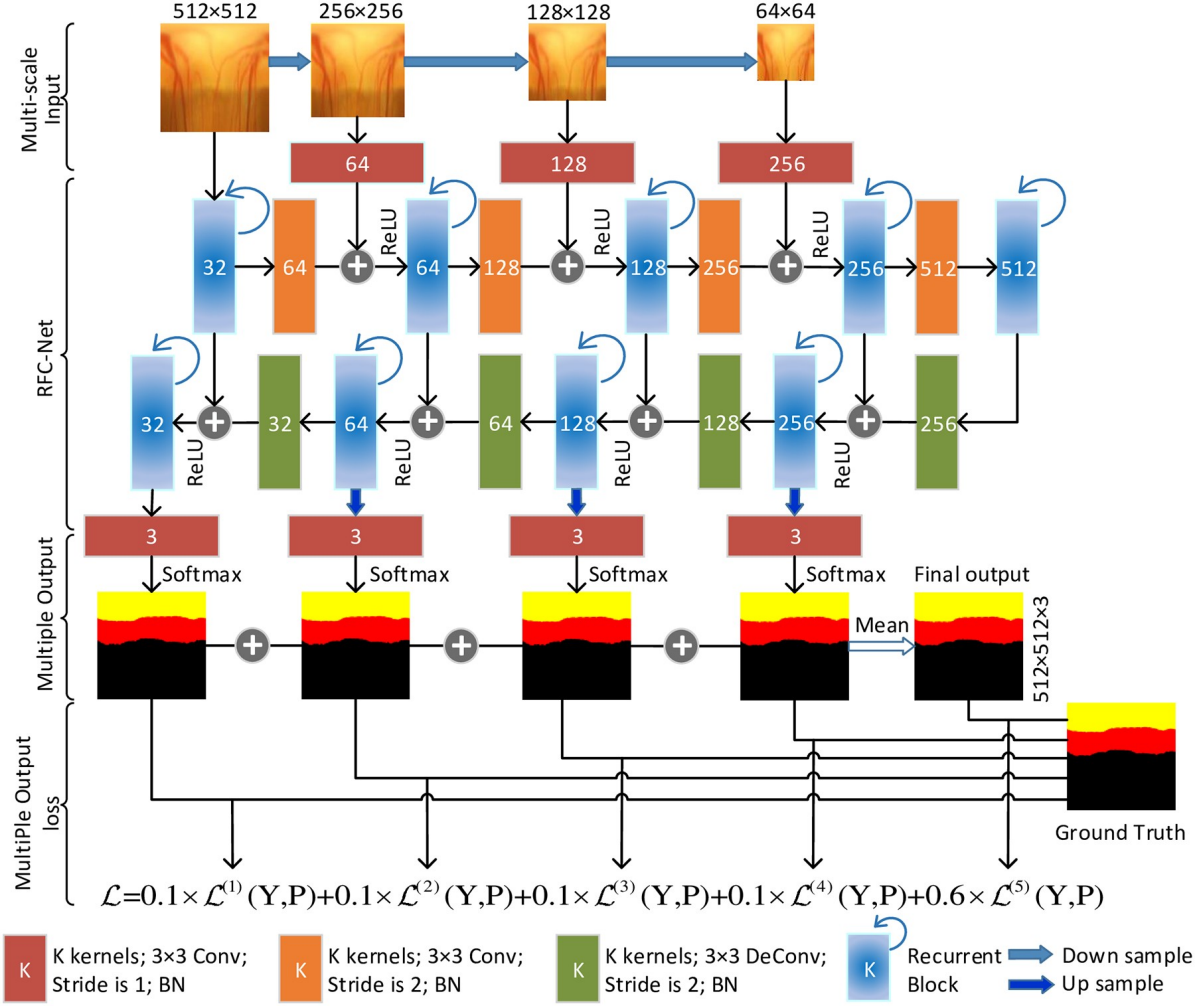
The structure of RFC-Net. Each blue block represents a recurrent block, each red block represents a 3 × 3 convolution, each orange block represents a 3 × 3 deconvolution.

Our proposed model RFC-Net is shown in Fig 3. Each blue bar represents a recurrent block, and each orange and green bar corresponds to a multi-channel feature map, the number in the bar represents the number of kernels. The orange bar uses 3 × 3 convolution, the green bar uses 3 × 3 deconvolution, and the arrows of different colors indicate different operations. First, we improved FCN. In order to solve the problem of insufficient correlation between the model’s receptive field and the features in the standard 3 × 3 convolution, we proposed a recurrent block to replace the standard 3 × 3 convolution. In addition, we tried to stack multiple layers of recurrent convolutions together to obtain a deeper recurrent architecture. Based on this, four variants of recurrent blocks were proposed: *RecurrentUnits*, *StackRecurrentUnits*, *RecurrentBasicUnits*, and *StackRecurrentBasicUnits*. Second, we use data preprocessing and polar transformation to alleviate the problems of model overfitting and low segmentation accuracy due to the small size of the medical dataset. Third, we replace the downsampling with a convolutional layer with asynchronous length. The resolution of the feature map after each convolution is reduced to 1/2 of the original feature map, alleviating the loss of feature information caused by downsampling. We use deconvolution for upsampling to restore feature information. We use “skip connection” to connect the recurrent block of the encoder part with the deconvolution of the decoder part, and add the corresponding features in the encoder to the corresponding layer in the decoder, which promotes the fusion of low-level information and high-level information. So that the segmentation feature map obtains complete context information. Forth, in order to speed up convergence and avoid model overfitting, batch normalization function (BN) is used to normalize the feature map of each layer after the convolution operation of each layer, and then using the ReLU activation function to activate it.

### 3.2 Multi-scale input

Multi-scale input has been shown to be successful in improving segmentation quality [29]. RFC-Net downsamples the image, then it builds a multiscale input in the encoder path. Downsampling a 512 × 512 image to form a 256 × 256 image, we can get a thumbnail of the original image. This process is repeated three times until the original image becomes a 64 × 64 image. Through this process, four types of images with different sizes can be obtained, which are 512 × 512, 256 × 256, 128 × 128, and 64 × 64. The four types of images obtained form a pyramid shape, as shown in Fig 3. By multi-scale input layer, a large increase in parameters is avoided effectively, and the network width of the decoder path is increased.

### 3.3 Recurrent block

**Fig 4:**
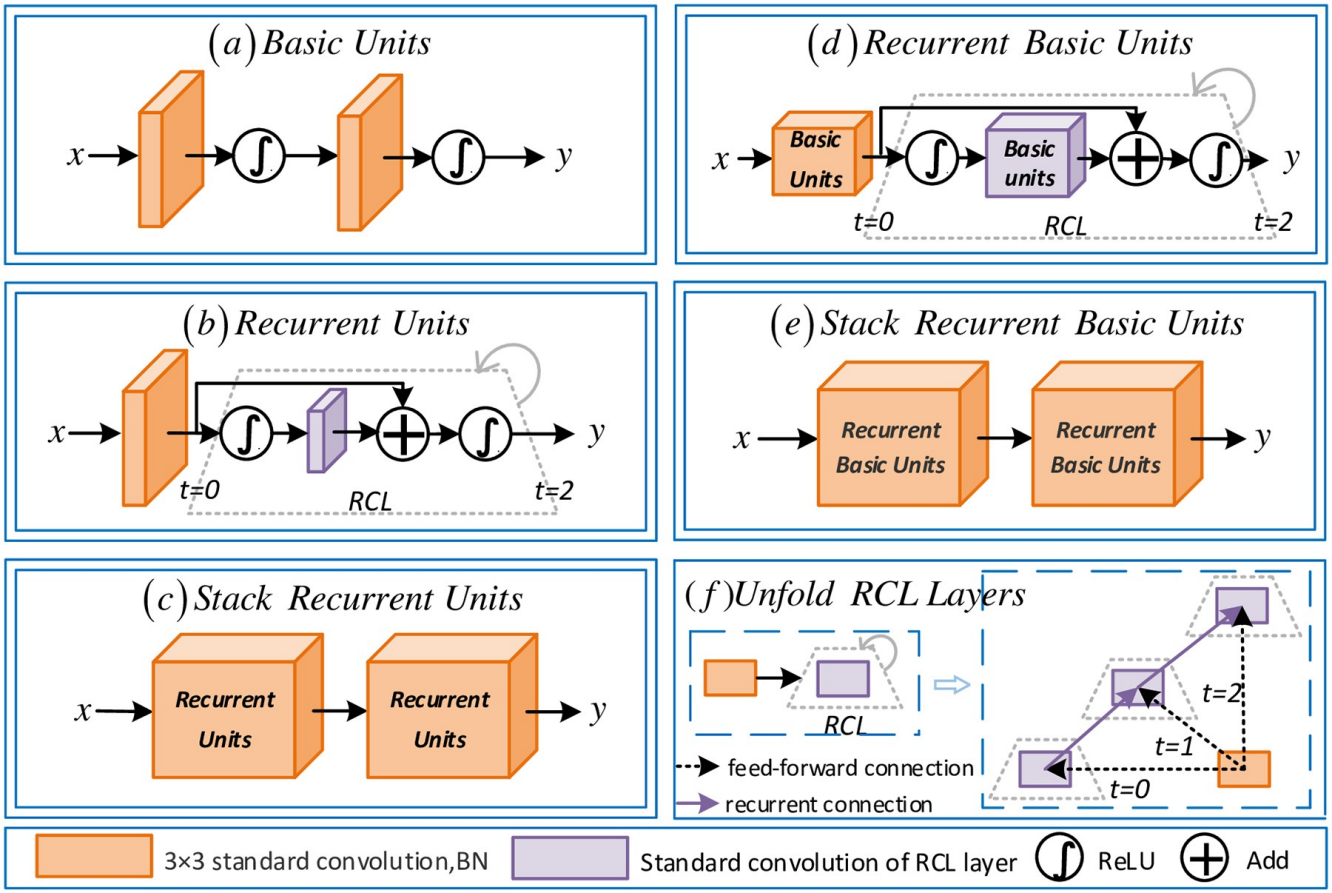
Different variant of standard convolutional and recurrent blocks. (a)Basic Units, (b)Recurrent Units, (c)Stack Recurrent Units, (d)Recurrent Basic Units, (e)Stack Recurrent Basic Units, (f)Unfold RCL Layers.

In this paper, based on RCNN [26], we designed a recurrent block. By combining the recurrent block into each convolutional layer, we make the network more abundant. The innovation of the recurrent block is that four different rekurrent convolution units are proposed. We applied these four different recurrent convolution units to RFC-Net respectively, and designed ablation experiments for verification in the experimental part. Fig 4 shows different variants of the standard convolution unit and the recurrent convolution units.

Fig 4 shows different variants of the standard convolutional unit and the recurrent convolution unit. Fig 4(a) is a standard convolution unit, named *BasicUnits*. The proposed four different recurrent convolution units are shown in Fig 4(b)–4(e). Fig 4(b) is named *RecurrentUnits* and includes a convolution layer and Recurrent Convolutional Layers (RCL) layer. Compared with the standard 3×3 convolution, the recurrent block uses RCL to extract image objects from the input layer. RCL does not directly output the input layer elements, but uses a variable recurrent network to process the data. It extracts the data twice and abstracts the elements. This attribute enhances the model’s ability to integrate contextual information, which is important for the edge detail segmentation of OD and OC. In addition, we try to stack *RecurrentUnits* together to obtain a deeper recurrent architecture. Based on this, *StackRecurrentUnits* is proposed, which is a stack of two *RecurrentUnits*, as shown in Fig 4(c). In Fig 4(d), we propose *RecurrentBasicUnits*. This unit modifies *RecurrentUnits* and replaces the standard convolutional layer with *BasicUnits* to further deepen the network. In Fig 4(e), we propose *StackRecurrentBasicUnits*, which is a stack of two *RecurrentBasicUnits*. In Fig 4(c) and 4(e), we verify the effect of deep recurrent networks by stacking recurrent layers together to obtain a deeper recurrent layer architecture. In this work we evaluated five different architectures.

The key to *RecurrentUnits* is the RCL layer. The state of the RCL layer develops on discrete time steps, and its unfolding structure is shown in Fig 4(f), where *t* = 2(0 ∼ 2) refers to the recurrent convolution operation. For the (*i*, *j*) unit located on the *k*_*th*_ feature map in the RCL, the net output $z_{k}^{\left(i,j\right)}\left(t\right)$ at time step *t* is given by:

$$\begin{array}{c} z_{k}^{\left(i,j\right)}\left(t\right)=\text{Sum}[W_{k}^{x}x_{k}^{\left(i,j\right)}\left(t-1\right),W_{k}^{r}r_{k}^{\left(i,j\right)}\left(t\right)]+b_{k} \end{array}$$ (1)

Among them, $x_{k}^{\left(i,j\right)}\left(t-1\right)$ and $r_{k}^{\left(i,j\right)}\left(t\right)$ represent feed-forward and recurrent input, respectively. They are vectorized patches centered on (*i*, *j*) on the *k*_*th*_ feature map in the previous layer and the current layer. sum[⋅] stands for element-wise summation. $W_{k}^{x}$ and $W_{k}^{r}$ represent vectorized feedforward and recurrent weights, respectively, and *b*_*k*_ is bias. The output $y_{k}^{\left(i,j\right)}\left(t\right)$ on the *k*_*th*_ feature map in RCL is fed to the standard ReLU activation function ∫ and expressed as:

$$\begin{array}{c} y_{k}^{\left(i,j\right)}\left(t\right)=\int (z_{k}^{\left(i,j\right)}\left(t\right))=\text{max}(z_{k}^{\left(i,j\right)}\left(t\right),0) \end{array}$$ (2)

In Fig 4(f), both feed-forward and recurrent connections we designed have local connections and shared weights among different locations. At *t* = 0, *t* = 1 and *t* = 2, the same convolution operation is used, and no additional convolution layer is added. The recurrent connection through weight sharing, it does not need to bring in additional parameters and calculations, and maintains the learning ability to further extract the edge detail information of OD and OC.

### 3.4 Polar transformation block

**Fig 5:**
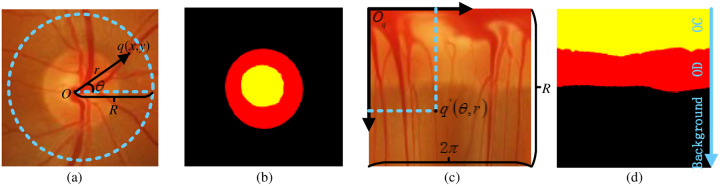
Polar transformation. (a) and (b) represent retinal images in cartesian coordinates. (c) and (d) represent retinal images in polar coordinates. In (b) and (d), where yellow represents OC, red represents OD, and black represents background.

Polar transformation is introduced to improve the performance of OD and OC segmentation. Fig 5(a) and 5(b) represent retinal images in cartesian coordinates. Fig 5(c) and 5(d) represent retinal images in polar coordinates. Fig 5(b) and 5(d) are the ground truth. Let *q*(*x*, *y*) denote the point on the plane of the fundus image, where the origin is set to the disc center *O*(*x*_*o*_, *y*_*o*_). Here, (*x*, *y*) takes the cartesian coordinate. *q*′(*θ*, *r*) takes the polar coordinate, *r* is the radius, *θ* is the directional angle. The polar transformation is defined as:

$$\begin{array}{c} \left\{ \begin{array}{c} x=r\text{cos}\theta \\ y=r\text{sin}\theta \end{array}\Leftrightarrow \{\begin{array}{c} r=\sqrt{x^{2}+y^{2}}\\ \theta ={\text{tan}}^{-1}\frac{y}{x} \end{array}\right. \end{array}$$ (3)

### 3.5 Multiple output cross entropy loss function

In our RFC-Net, we introduce the multiple output layers. The advantage of the multiple output layer is that it can backpropagates the loss of each layer output and the loss of the final layer output to the early layer in the decoder path, which not only effectively alleviates the gradient vanishing problem, but also helps to train the model. We apply multipel output cross entropy loss function for each multiple output image. Let’s write *X* = {*x*_*i*_, *i* = 1, …, *N*} for the original set of input image. *N* for the number of pixels. *Y* = {*y*_*i*_, *i* = 1, …, *N*} for the corresponding ground truth of each input image, *y*_*i*_ ∈ {0, 1, 2}. Where *O* is the number of categories, *O* = 3, 0 corresponds to background, 1 corresponds to OD, and 2 corresponds to OC. *V* for the probalility, the probability that the *i*_*th*_ sample is predicted to be the *o*_*th*_ category is *v*_*i*,*o*_.

Assuming that there are *M* multiple output layers in the network, the corresponding loss weights for each multiple output layer are expressed as *α*_*i*_ = {*α*_*i*_, *i* = 1, …, *M*}. Here, *M* = 5, and *α*_*i*_ = {0.1, 0.1, 0.1, 0.1, 0.6}. For each multiple output image, the loss $L^{\left(i\right)}$ is calculated separately. $L^{\left(i\right)}$ is defined as:

$$\begin{array}{c} L^{\left(i\right)}\left(Y,V\right)=-\frac{\alpha_{i}}{N}\sum_{i=0}^{N-1}\sum_{o=0}^{O-1}y_{i,o}{\text{log}}_{vi,o} \end{array}$$ (4)

We superimpose the $L^{\left(M\right)}$ of each multiple output layer. The final output loss function is L, L is defined as:

$$\begin{array}{c} L=\sum_{i=0}^{M-1}L^{\left(i\right)}\left(Y,V\right) \end{array}$$ (5)

## 4 Experiments and analysis

We made our experiment on the Drishti-GS1 dataset [30]. The training set contains 50 images and the testing set contained 51 images. The OD and OC area of all images are marked by 4 ophthalmologists with different clinical experience, we trained the average area marked by 4 experts as a standard OD and OC area.

The number of images in the Drishti-GS1 dataset is limited. In response to this problem, we have designed effective data preprocessing, which can not only expand the number of training samples, but also increase the diversity of training samples. First, we use the YOLOv2 [28] model to extract the OD image. Then perform data expansion on the detected image. Images of different sizes are taken based on the center point of the OD, including 400 × 400, 500 × 500, 550 × 550, 600 × 600, 650 × 650, 700 × 700, 750 × 750, 800 × 800, 850 × 850, and 900 × 900. These images are used to enhance the data. The size of the input image is scaled to the standard 512 × 512 when training the network. On the one hand, this data preprocessing method can avoid overfitting, and on the other hand, it will bring about the improvement of model performance.

### 4.1 Implementation

We implemented the RFC-Net using the PyTorch deep learning framework [31]. The hardware environment of our laptops includes NVIDIA GeForce GTX 1060 GPU, Intel Core i7-7700HQ CPU@2.80 GHz processor, 32 GB of RAM, and running Linux Ubuntu OS 16.04. All training and testing were performed in the same hardware environment. During the training, RFC-Net uses the Stochastic gradient descent (SGD) optimizer [32]. In our experiment, the training is iterated for a total of 400 epochs. On the setting of the learning rate *lr*, *lr* is initialized to 0.0001, the weight attenuation coefficient is 0.0005, and the momentum is 0.9. At the same time, we used the learning rate scheduler, which can achieve good learn preformance without complicated fine-tune the learning rate. Taking 2 sample input models randomly from the training set {*x*_*i*_, *y*_*i*_}, this can reduce the instability of the stochastic gradient. Convolution is used to extract features and restore images using ReLU as an activation function. The size of the output segmentation image is 512 × 512 × 1. For more details on implementation please refer to our code and logs at https://github.com/HaiCheung/RFCN.

### 4.2 Measurement of the classificaiton performance

In order to measure the classification performance of the OD/OC segmentation system, we compared the Sensitivity(SEN), Specificity(SPC), Accuracy(ACC), F1 and boundary distance localization error (BLE) [30]. Sensitivity(SEN), Specificity(SPC) and Accuracy(ACC) are defined as:

$$\begin{array}{c} SEN=\frac{TP}{P} \end{array}$$ (6)

$$\begin{array}{c} SPC=\frac{TN}{N} \end{array}$$ (7)

$$\begin{array}{c} ACC=SEN\times \frac{P}{\left(P+N\right)}+SPC\times \frac{N}{\left(P+N\right)} \end{array}$$ (8)

Measurements of F1, Precision and Recall are also widely used in classification, which are defined as:

$$\begin{array}{c} F1=2\times \frac{Precision\times Recall}{Precision+Recall} \end{array}$$ (9)

$$\begin{array}{c} Precision=\frac{TP}{TP+FP} \end{array}$$ (10)

$$\begin{array}{c} Recall=\frac{TP}{TP+FN}=\frac{TP}{P} \end{array}$$ (11)

Among them, TP, TN, FP, FN, P and N represent true positive, true negative, false positive, false negative, positive samples and negative samples, respectively.

Similarly, BLE is used to evaluate the boundary distance (in pixels) between the edge (*C*_0_) of the model segmentation result *U*(*x*) and the edge (*C*_*g*_) of *y*. BLE is better able to embody the local (boundary) level of segmentation, which are used by the [19, 20]. It is defined as:

$$\begin{array}{c} BLE\left(C_{0},C_{g}\right)=-\frac{1}{N}\sum_{\theta =0}^{N-1}\sqrt{{\left(d_{g}^{\theta }\right)}^{2}-{\left(d_{o}^{\theta }\right)}^{2}} \end{array}$$ (12)

Here, $d_{g}^{\theta }$, $d_{o}^{\theta }$ denotes the euclidean distance between *C*_*g*_, *C*_0_ and the center point in the direction of *θ*, respectively, with 24 equidistant points being considered (*N* = 24). The desirable value for BLE is 0.

### 4.3 Comparative experiment and analysis before and after polar transformation

During the training phase, we test the effects of data augmentation and polar transformation on model segmentation.

#### Data Augmentation(DA)

For each fundus image, the following preprocessing is carried out, including random horizontal flip, random vertical flip, random rotation within the range of [0°, 360°], and random cropping. For a image sized in 512 × 512, it is cut out randomly by filling 64 pixels on the top, bottom, left and right of each picture.

#### Polar Transformation(PT)

By adding polar transition in the network, the effect of using polar transformation on model segmentation accuracy is verified.

We apply the *BasicUnits* proposed in Fig 4(a) to RFC-Net and use it as the main network structure of the experiment. The results of DA and PT for the OD and OC segmentation are compared, which are shown in Table 1. In Table 1, the experiment results showing that DA does not make help to OD and OC segmentation, while PT contriubtes a lot. In the OD and OC segmentation results, compared with the results without DA and PT, the F1 score of PT is increased 3.43% and 15.55%, the BLE is reduced by 5.02 pixels and 9.84 pixels. By applying PT, it helps to avoid over-fitting during model training and further improve the segmentation performance. Therefore, PT is applied in all of the following experiments.

**Table 1:** Segmentation results with/without DA and PT.

Method	Parament	OD(mean/std)		OC(mean/std)	
		F1	BLE(px)	F1	BLE(px)
−	11016684	0.9413/0.018	11.24/3.51	0.7327/0.031	25.38/18.57
*DA*	11016684	0.9192/0.113	18.54/17.45	0.7982/0.189	26.71/16.97
*PT*	11016684	**0.9756/0.013**	**6.22/3.29**	**0.8882/0.116**	**15.54/6.96**
*DA* + *PT*	11016684	0.9735/0.011	6.27/3.32	0.8819/0.079	15.94/6.77

**Fig 6:**
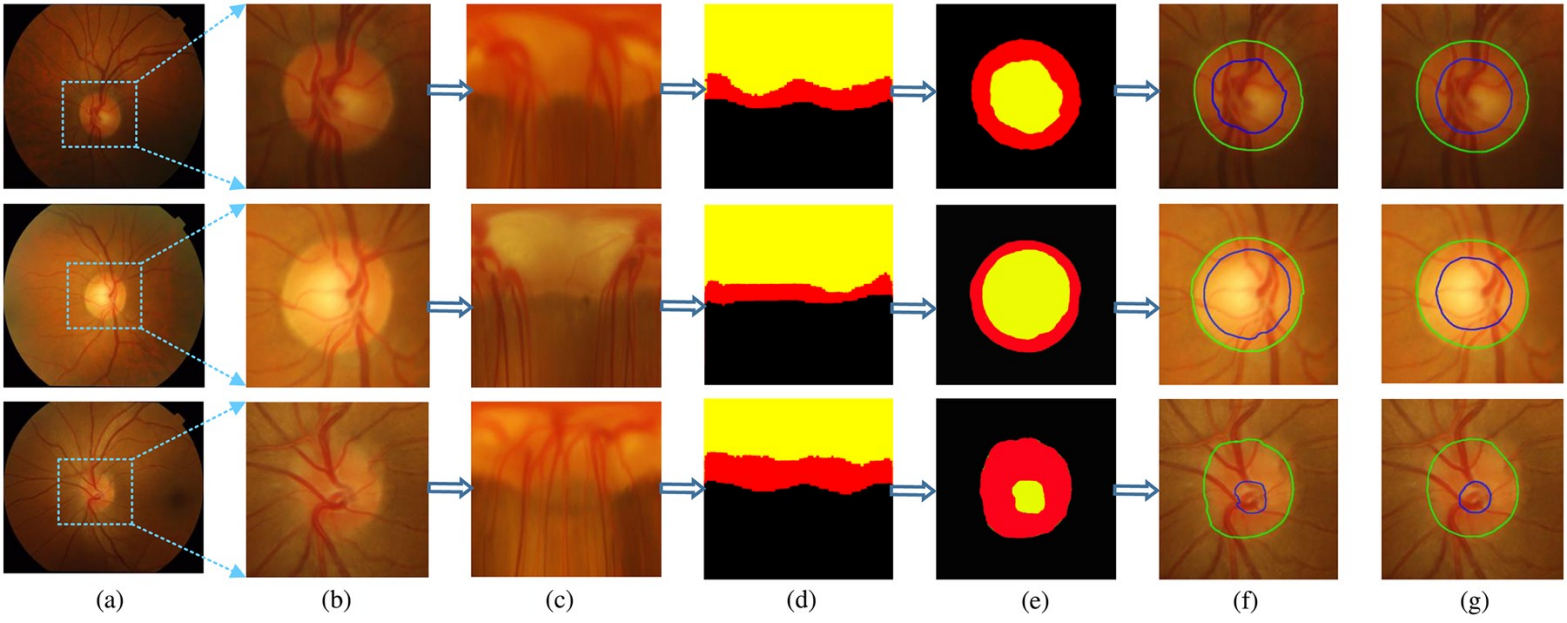
Visualization of the segmentation results before and after using polar transformation. (a) Fundus retinal image. (b) The optic disc image extracted from (a). (c) The image after polar transformation. (d) Segmented image in polar coordinate system. (e) Segmented image in cartesian coordinate system. (f) Restore the segmented image under the optic disc image from (e). (g) Ground Truth. The green contour represent the boundary of OD, the blue contour represent the boundary of OC.

We visually show the changes in the retinal image before and after the polar transformation and the segmentation curve. Fig 6 shows the edge curves of the joint OD and OC segmentation of polar transformation in samples drishtiGS 6, drishtiGS 7, and drishtiGS 100. From Fig 6, we can see two main advantages of polar transformation: (i) Expansion of the OC ratio: Polar transformation increases the OC ratio. Taking the sample drishtiGS 100 as an example, the area of OC in Fig 6(e) is only 4.7%. While in Fig 6(d), the area of OC increases to 24.5%. This will make the area of the OC, OD and background more balanced and greatly assist in segmenting OD and OC. By balancing the area between the OC, OD and background, not only can avoid over-configuration when training the model, but also increase the accuracy of segmenting the OD and OC. (ii) Clearer the space constraints between the OD and the OC: In the original fundus image, the redial relationship between the OD and the OC should be that the OC is inside the OD area, as shown in Fig 6(b). However, this redial relationship is difficult to achieve in the original cartesian coordinate system. The polar transformation shifts this redial relationship to the spatial relationship, as shown in Fig 6(d). Among them, the area of OC, OD, and background shows an ordered layer structure, this layer structure is convenient to use, especially for the segmentation of OD and OC, and the effect is significant.

We further verified the effectiveness of polar transformation from a statistical point of view, it would make the results more convincing. Taking the *BasicUnits* proposed in Fig 4(a) as an example, we propose a hypothesis: The performance of *BasicUnits* using polar transformation is better than *BasicUnits* not using polar transformation. We conducted a *P* − *value* analysis of the F1 indicators and the results are shown in Table 2. The statistical method used SPSS19.0 software to analyze the data, and *P* − *value* < 0.05 indicated that the difference was statistically significant. *BasicUnits*+ *PT*: *BasicUnits* represents the comparison between the *BasicUnits* using polar transformation and the *BasicUnits* without polar transformation. In Table 2, the *P* − *value* of *BasicUnits* + *PT*: *BasicUnits* is less than 0.05 for OD and OC, which indicates that the *BasicUnits* + *PT* and *BasicUnits* have statistical significance. In other words, the performance of the *BasicUnits* using polar transformation is better than the *BasicUnits* without polar transformation. Therefore, in the subsequent experiments, we all carried out on the basis of adding polar transformation.

**Table 2:** Comparison of *P* − *value* analysis results based on F1 indicators.

	OD	OC
	*BasicUnits* + *PT*: *BasicUnits*	*BasicUnits* + *PT*: *BasicUnits*
*P* − *value*	0.007	0.003

### 4.4 Comparison of results before and after model improvement

In order to verify the efficiency of the proposed structure to improve the model, the effect of the OD and OC segmentation with/without the proposed structure is compared with the different network structures used in this paper. The five structures which proposed in Fig 4 are taken into the RFC-Net of Fig 3 for experiments. The experimental results are shown in Tables 3, 4 and 5.

**Table 3:** Experiment results at F1 and BLE for the recurrent block in the Fig 4.

Recurrent Block	Parament	OD(mean/std)		OC(mean/std)	
		F1	BLE(px)	F1	BLE(px)
*BasicUnits*	11016684	0.9756/0.013	6.22/3.29	0.8882/0.116	15.54/6.96
*RecurrentUnits*	11017628	0.9761/0.014	4.43/2.63	0.8861/0.109	15.39/10.64
*StackRecurrentUnits*	18883436	**0.9787/0.009**	**3.96/1.79**	**0.9058/0.118**	15.40/11.19
*RecurrentBasicUnits*	18877548	0.9757/0.011	4.52/2.04	0.8834/0.103	**14.37/9.29**
*StackRecurrentBasicUnits*	34599276	0.9781/0.009	4.59/2.56	0.8908/0.094	15.48/11.26

**Table 4:** Experiment results at ACC, SEN and SPC for the recurrent block in the Fig 4.

Recurrent Block	Parament	OD			OC		
		ACC	SEN	SPC	ACC	SEN	SPC
*BasicUnits*	11016684	0.9761	0.9217	0.9780	0.9775	0.9625	0.9793
*RecurrentUnits*	11017628	0.9759	0.9374	0.9784	0.9774	0.9533	0.9788
*StackRecurrentUnits*	18883436	**0.9764**	**0.9578**	**0.9787**	**0.9778**	**0.9782**	0.9787
*RecurrentBasicUnits*	18877548	0.9762	0.9567	0.9783	0.9777	0.9687	**0.9794**
*StackRecurrentBasicUnits*	34599276	0.9763	0.9293	0.9788	0.9775	0.9511	0.9790

**Table 5:** Joint segmentation results at ACC, SEN and SPC for the recurrent block in the Fig 4.

Recurrent Block	Parament	Joint Optic Disc and Cup			
		ACC	SEN	SPC	F1(mean/std)
*BasicUnits*	11016684	0.9792	0.9548	0.9800	0.9318/0.009
*RecurrentUnits*	11017628	0.9793	0.9629	0.9798	0.9312/0.101
*StackRecurrentUnits*	18883436	**0.9795**	**0.9632**	**0.9801**	**0.9423/0.007**
*RecurrentBasicUnits*	18877548	0.9793	0.9592	0.9799	0.9296/0.107
*StackRecurrentBasicUnits*	34599276	0.9794	0.9598	0.9801	0.9346/0.106

Tables 3 and 4 show the segmentation results of OD and OC on the five evaluation indicators of F1, BLE, ACC, SEN and SPC. Table 5 shows the segmentation results of the joint OD and OC. In Table 3, by comparing the experimental results of these five structures, it is found that the RFC-Net using *StackRecurrentUnits* has the best effect on the segmentation of OD and OC. Both F1 and BLE evaluation indicators are better than the other four structures of RFC-Net. We observed that *StackRecurrentUnits* achieved the highest F1 score and the lowest BLE on OD, indicating that it can more accurately subdivide background, OD and OC. Compared with *BasicUnits*, F-measure increased by 0.31%, and BLE decreased by 2.26 pixels. This proves the effectiveness of *StackRecurrentUnits*. In Table 4, ACC, SEN and SPC of *StackRecurrentUnits* reached the highest on OD and OC. In the segmentation results of OD and OC, SEN is 3.61% and 1.57% higher than *BasicUnits* respectively. In Table 5, the segmentation results of the joint OD and OC also illustrate the effectiveness of *StackRecurrentUnits*. Through the experimental results, it can be seen that the recurrent block improves the model’s ability to understand local context information and maintains the relevance of feature information in the receptive field, so that the RFC-Net model can more accurately segment OD and OC. In the recurrent block, the segmentation effect of *StackRecurrentUnits* is the best. Because *StackRecurrentUnits* contains two *RecurrentUnits*, it further uses the role of *RecurrentUnits* to better capture local features and enrich contextual relevance. Therefore, in future work, we will conduct analysis and research based on *StackRecurrentUnits*.

By analyzing the data in Tables 3 and 4, we found that *BasicUnits* have insufficient ability to extract features, and the segmentation performance of OD and OC is low. For *RecurrentUnits*, we added RCL, and the segmentation effect is better than *BasicUnits*. This proves that we add the recurrent convolution is the correct choice. Because we use weight sharing in *RecurrentUnits*, compared to *BasicUnits*, the number of parameters of *RecurrentUnits* has basically not increased. For *RecurrentBasicUnits*, the segmentation effect of this structure is not as effective as *StackRecurrentUnits*. This shows that the method we designed to stack *RecurrentUnits* is effective, which can make the model learn highly complex features, which is very effective for the edge detail segmentation of OD and OC. Note that the structure of *StackRecurrentBasicUnits* is more complex and the network is deeper, but the segmentation performance is not as good as *StackRecurrentUnits*. We attribute this fact to the difficulty of learning such a deep network model. Therefore, we conclude that the sensitivity of standard 3 × 3 convolution to weight changes can better adjust the gradient, and recurrent convolution can better capture local features and enrich contextual relevance. However, if the number of recurrent layers is too deep, the network may learn redundant features in continuous convolutions and the problem of gradient dissipation will occur during the training process, resulting in reduced segmentation performance. By applying *StackRecurrentUnits* to the RFC-Net network structure, we can more accurately segment the fundus OD and OC images.

**Fig 7:**
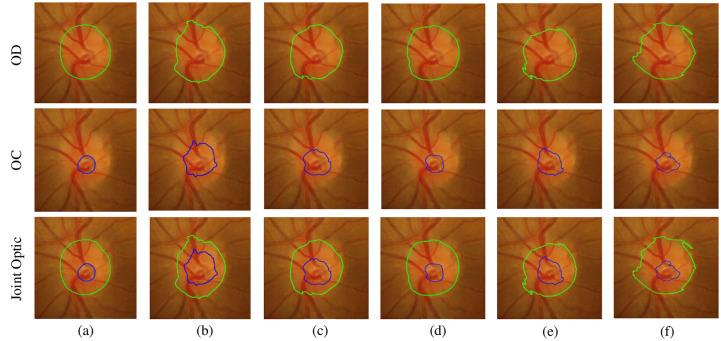
Comparison of segmented images with different recurrent units in the Drishti-GS1 dataset. (a) Ground Truth. (b) Basic Units. (c) Recurrent Units. (d) Stack Recurrent Units. (e) Recurrent Basic Units. (f) Stack Recurrent Basic Units. The green contour represent the boundary of OD, the blue contour represent the boundary of OC.

Fig 7 shows a visualization example of the OD, OC and joint OD and OC segmentation results of the five structures in the sample drishtiGS 100. DrishtiGS 100 is a retinal image of a glaucoma case. It can be seen from Fig 7 that compared with *BasicUnits*, *StackRecurrentUnits* significantly improves the accuracy of the OD and OC segmentation results, which are basically the same as the segmentation area of Ground Truth, and the edge part of the segmentation result is smoother. Among them, the OC area segmented by *BasicUnits* is larger than Ground Truth, which will make the CDR value too large, and it is easy to be misjudged as glaucoma. After using *StackRecurrentUnits*, the segmented OC area is basically close to Ground Truth, which can effectively reduce this misjudgment. This proves that on the RFC-Net model, *StackRecurrentUnits* has better feature representation ability than other structures.

**Fig 8:**
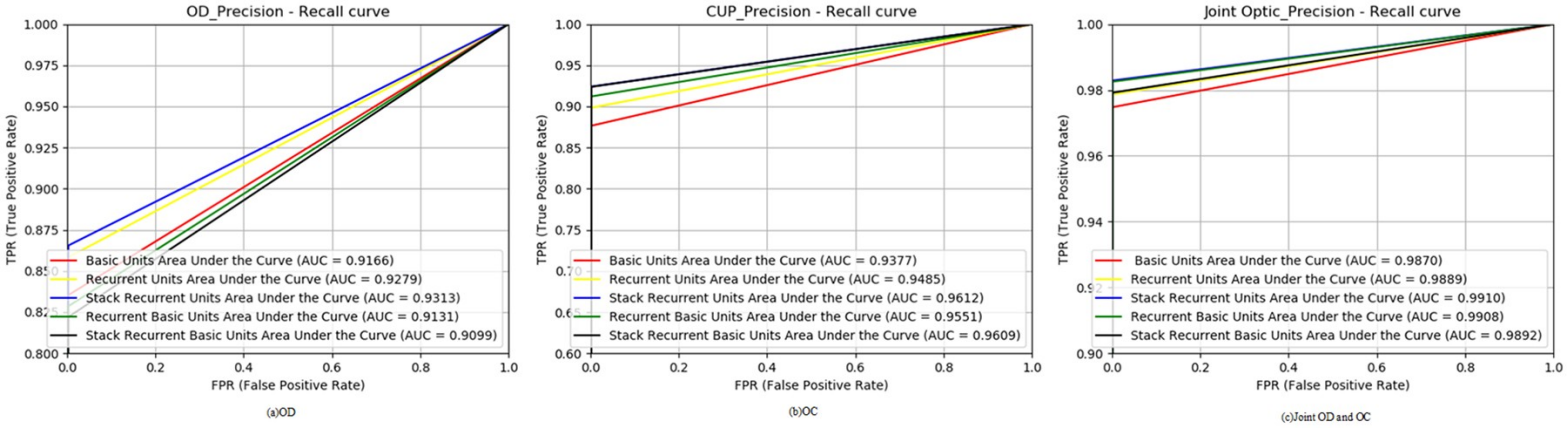
Comparison of Receiver Operating Characteristic (ROC) curves of each structure in the Drishti-GS1 dataset. (a) Cup. (b) Disc. (c) Optic.

To further illustrate the effectiveness of *StackRecurrentUnits*, we compared the ROC curves of the five structures in OD, OC, and joint OD and OC segmentation, as shown in Fig 8. *StackRecurrentUnits* has an increase in the area under the curve (AUC) of the optic disc by 0.0147 compared with *BasicUnits*, *StackRecurrentUnits* increased the AUC for the optic cup by 0.0235. The AUC of *StackRecurrentUnits* for joint optic disc segmentation was 0.9910. In summary, it can be shown that *StackRecurrentUnits* is effective in OD and OC segmentation. It can also be seen that it is reasonable to combine convolutional networks and recurrent networks. At the same time, it also shows that the OD and OC segmentation performance is also improved by adding recurrent block to the network.

### 4.5 Comparison of segmentation performance

In order to further demonstrate the effectiveness of RFC-Net for OD and OC segmentation, on the Drishti-GS1 dataset, in this section, we compare the performance of the proposed method with BCRF [18], Superpixel [15], and Graph cut prior [12], Boosting CNN [16], U-Net [6], RACE-Net [19], Stack-U-Net [20], pOSAL [33] and several other state-of-art OD and OC segmentation methods.

In our experiment, 51 fundus images in the testing set are segmented. As shown in Table 6, we show the segmentation results of the RFC-Net in F1 and BLE. Compared with other recent methods, RFC-Net achieves the best performance in both F1 and BLE. BCRF [18] has jointly segmented the disc and the cup based on the Conditional Random Field, and obtained the most advanced performance on OD segmentation. However, its performance for OC segmentation is not good enough. In [12], this algorithm regards OD and OC segmentation as a pixel labeling problem, the depth information is not considered by the algorithm. Hence, the segmentation accuracy is not accurate enough. In Boosting CNN [16], its ability for feature extraction is weak, and deeper semantic information cannot be learned. Therefore, the performance for OD and OC segmentation is relatively poor. In Superpixel [15] algorithm, because it is based on various hand-made visual features, discriminating representations are not enough, so it is easily affected by the lesion area. For U-Net [6], there is a lack of receptive field, and the global context information of the fundus image cannot be fully understood, so that the segmentation effect on the OC is not ideal. In RACE-net [19] algorithm, insufficient feature extraction for fundus images is carried out without strong intensity gradient, and it results in poor segmentation performance. Stack-U-Net [20] improved the structure proposed in [6], but the number of model parameters increased linearly with the increase of the number of blocks, and the accuracy of the OD and OC segmentation is not as good as the proposed method. In pOSAL [33] algorithm, pOSAL framework focus is on enhancing the robustness of the deep network through domain shift, ignoring the relationship between the OD and the OC, and the edge information of the OC cannot be accurately extracted. The segmentation accuracy is not good enough. Through extensive experimental evaluation and comparison with existing methods, it is shown that the proposed RFC-Net framework is superior to most recent methods for the OD and/or OC segmentation. Our approach captures edge detail information more efficiently and learns better feature representations in the OD and OC segmentation.

**Table 6:** Experiment results of OD and OC segmentation on Drishti-GS1 dataset.

Author	Method	Year	OD(mean/std)		OC(mean/std)	
			F1	BLE(px)	F1	BLE(px)
*JoshiGD* [34]	Multiview	2012	0.9600/0.020	8.93/2.96	0.7900/0.18	25.28/18.00
*ChengJ* [15]	Suprpixel	2013	0.9500/0.020	9.38/5.75	0.8000/0.140	22.04/12.57
*ZhengY* [12]	Graph cut prior	2013	0.9400/0.060	14.74/15.66	0.7700/0.160	26.70/16.67
*ZillyJG* [16]	Boosting CNN	2015	0.9470/0.030	9.10/3.10	0.8300/0.140	16.50/11.01
*RonnebergerO* [6]	U-Net	2015	0.9600/0.020	7.23/4.51	0.8500/0.100	19.53/13.98
*Sevastopolsky* [17]	Modication-U-Net	2017	0.9500/-	-/-	0.8500/-	-/-
*ChakravartyA* [18]	BCRF	2017	0.9700/0.020	6.61/3.55	0.8300/0.150	18.61/13.02
*ArunavaC* [19]	RACE-net	2018	0.9700/0.020	6.06/3.84	0.8700/0.090	16.13/7.63
*SevastopolskyA* [20]	Stack-U-Net	2018	0.9700/-	-/-	0.8900/-	-/-
*Al* − *BanderB* [7]	DenseNet FCN	2018	0.949/-	-/-	0.8282/-	-/-
*WangS* [33]	pOSAL	2019	0.965/-	-/-	0.858/-	-/-
*Proposed*	RFC-Net	-	**0.9787/0.009**	**3.96/1.79**	**0.9058/0.118**	**15.40/11.19**

**Fig 9:**
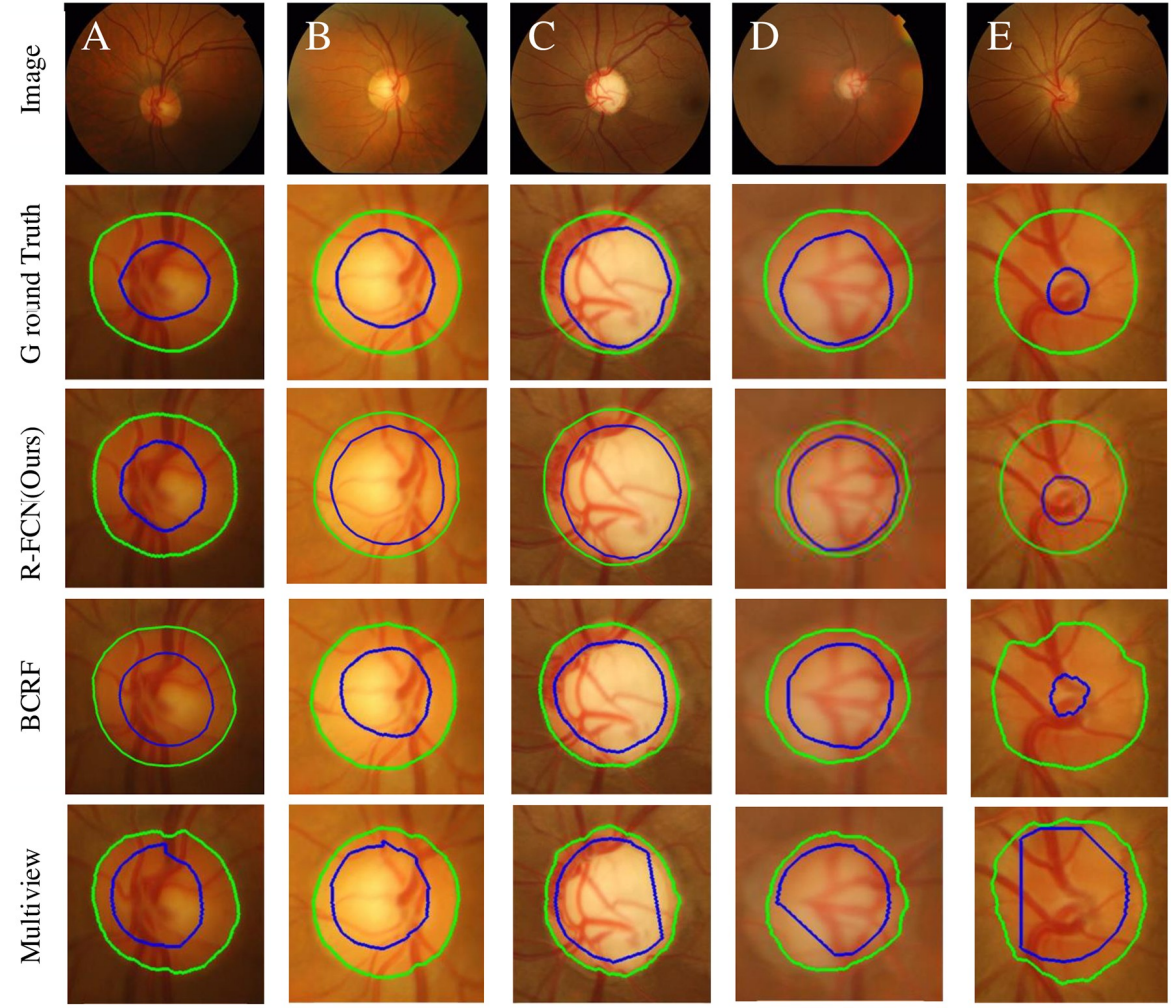
This results demonstrates qualitative results of the proposed RFC-Net. First column is the original image, second column is the ground truth, third column is the results of the RFC-Net(ours), four column is the results of the BCRF [18], and five column is the results of the Multiview [34]. The green contour represent the boundary of OD, the blue contour represent the boundary of OC.

Fig 9 shows the edge curve between the OD and OC segmented in the sample drishtiGS 006, drishtiGS 007, drishtiGS 019, drishtiGS 05 and drishtiGS 100 by several sate-of-art methods, including BCRF [18] and Multiview [35]. The edge curve of other algorithms are omitted here for sake of space. It is shown in Fig 9 that the RFC-Net model proposed divides the OD and OC boundary better than other methods. Regardless of the fundus image of a normal person or a patient, the error between the edge of the segmented area and the edge of the standard area is quite small, especially for the segmentation of the OC, which helps to validate the proposed algorithm.

### 4.6 Quantitative analysis of segmentation results of different competitive algorithms

The proposed RFC-Net model shows excellent performance in OD and OC segmentation. In order to make our results more convincing, we have selected several more competitive algorithms and tested them in the experimental environment described in this paper. We used 51 test images on the Drishti-GS1 dataset, and tested them on FCN [5], U-Net [6], M-Net [21] and CE-Net [23] respectively.

When experimenting on FCN [5], U-Net [6], M-Net [21] and CE-Net [23] in this paper, our training parameter settings are as follows: (1) We reproduce FCN [5], U-Net [6], M-Net [21] and CE-Net [23] only set random initial weights. (2) We set the batch to 8, train on NVIDIA Tesla K80 (12G) GPU, use Python 3.6 as the programming language, use Pytorch 1.0.0 deep learning framework for algorithm design and coding, and use Nesterov momentum Stochastic gradient descent method for end-to-end training.

As shown in Table 7, with DRISHTI GS1, the RFC-Net model can segment the OD regions with around 0.9787, 3.96, 0.9764, 0.9578, and 0.9778 of F1, BLE, accuracy, sensitivity and specificity, respectively, the RFC-Net model can segment the OC regions with around 0.9058, 15.40, 0.9778, 0.9782, and 0.9787 of F1, BLE, accuracy, sensitivity and specificity, respectively. The experimental results show that the five evaluation indicators of RFC-Net are better than other network models. Compared with the latest CE-Net, the F1 score of our method is increased by 3.59% in the OC segmentation, which is a great improvement and effectively proves that the RFC-Net model has better performance.

**Table 7:** F1, BLE, accuracy, sensitivity and specificity with the RFC-Net, FCN, U-net, M-Net and CE-Net models on Drishti-GS1 dataset.

Method	OD					OC				
	F1	BLE	ACC	SEN	SPC	F1	BLE	ACC	SEN	SPC
*FCN* [5]	0.9321	8.90	0.9149	0.9021	0.9475	0.8170	21.83	0.9217	0.9176	0.9515
*U* − *Net* [6]	0.9600	7.23	0.9579	0.9417	0.9579	0.8500	19.53	0.9493	0.9592	0.9678
*M* − *Net* [21]	0.9621	6.07	0.9619	0.9512	0.9678	0.8513	17.96	0.9682	0.9622	0.9711
*CE* − *Net* [23]	0.9688	5.04	0.9714	0.9567	0.9614	0.8699	16.06	0.9725	0.9671	0.9715
*RFC* − *Net*	**0.9787**	**3.96**	**0.9764**	**0.9578**	**0.9783**	**0.9058**	**15.40**	**0.9778**	**0.9782**	**0.9787**

**Fig 10:**
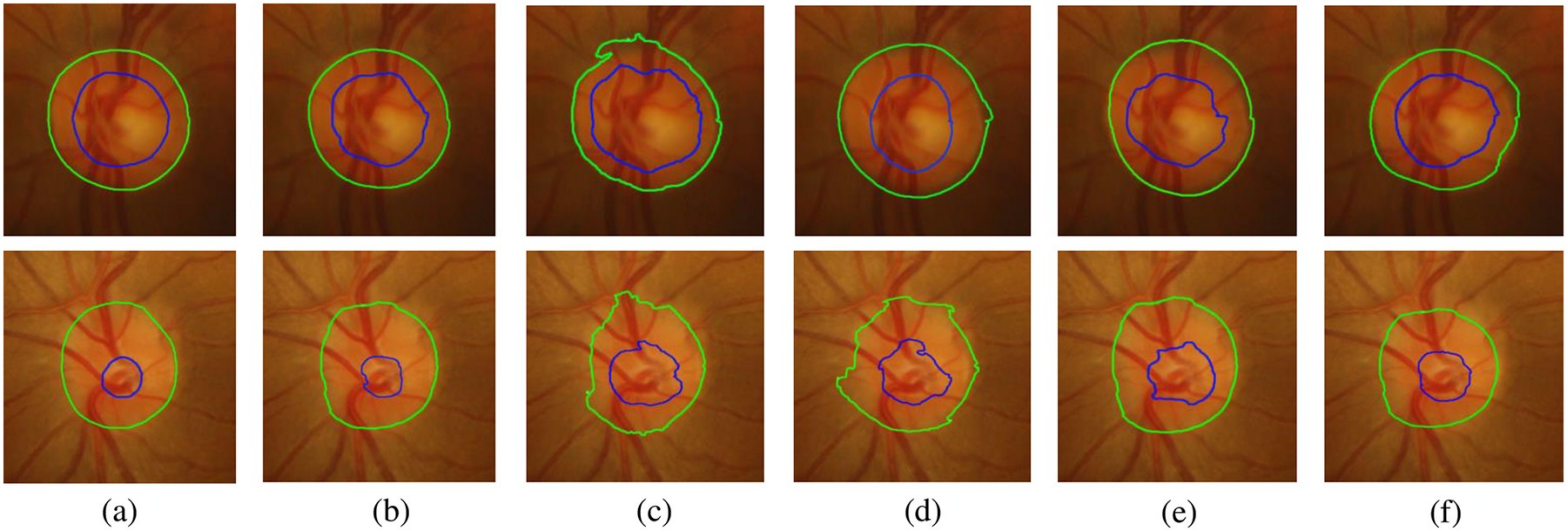
Ground truth, our methods RFC-Net, FCN, U-Net, M-Net and CE-Net real segmentation contours. (a) Ground Truth. (b)RFC-Net. (c)FCN. (d)U-Net. (e)M-Net. (f)CE-Net.

In order to show the segmentation effect of OD and OC more clearly, we select a normal eye image and a glaucoma image respectively, and compare the real segmentation contours of FCN [5], U-Net [6], M-Net [21], CE-Net [23] and our method. As shown in Fig 10, compared with the four competitive algorithms, our model has a clearer segmentation boundary, and the segmentation curves of OD and OC are closest to Ground Truth. It is worth noting that our model has obvious advantages for OC segmentation. It can be seen that our proposed RFC-Net can greatly improve the performance of lesion segmentation.

## 5 Discussion

### 5.1 Is module fusion effective?

Our method jointly divides the OD and OC regions and considers their correlation in polar coordinates. In RFC-Net, in order to prove the effectiveness of the proposed recurrent module, polar module, multi-scale input module and multiple output module, we performed the following ablation studies on the Drishti-GS1 dataset:

#### Ablation studies using improved FCN

Our proposed method is based on FCN, so FCN is the most basic benchmark model. We improved the basic FCN model, using 3 × 3 convolution instead of downsampling to further learn feature map semantic information and position information, and transposed 3 × 3 convolution for performing upsampling to obtain refined edges. Four proposed recurrent units are the contribution to this paper, they have been analyzed and compared in Table 8. Here we select the *StackRecurrentUnits* in the Fig 4(c) with the best effect and replace the convolution in the basic FCN with the *StackRecurrentUnits* to enhance the learning ability. We call the improved FCN network with *StackRecurrentUnits* as ‘Backbone’. We also performed experiments to compare the segmentation results of Backbone with the basic FCN. Table 8 shows the segmentation results of the two methods. As we can see, compared with the segmentation results of the basic FCN. On the OD, the F1 score of the backbone increased by 0.92%, and the BLE decreased by 2.29. On the OC, the F1 score of the backbone increased by 1.3%, and BLE decreased by 3.22. The results show that we are effective in improving the basic FCN.

**Table 8:** Ablation study of each module on Drishti-GS1 dataset.

Method	OD(mean/std)		OC(mean/std)	
	F1	BLE(px)	F1	BLE(px)
*FCN* [5]	0.9321/0.102	8.90/5.74	0.8170/0.103	21.83/15.67
*Backbone*	0.9413/0.020	6.61/3.55	0.8300/0.150	18.61/13.02
*Backbone* + *Input* + *Output*	0.9516/0.119	6.23/3.51	0.8500/0.100	18.11/13.70
*Backbone* + *PT*	0.9600/0.020	5.19/3.95	0.8700/0.090	16.13/12.63
*M* − *Net* [21]	0.9621/0.170	6.07/4.71	0.8513/0.192	17.96/14.05
*CE* − *Net* [23]	0.9688/0.003	5.04/3.69	0.8699/0.117	16.06/13.11
*U* − *Net* [6]	0.9600/0.020	7.23/4.51	0.8500/0.100	19.53/13.98
*U* − *Net* + *PT* + *StackRecurrentUnits*	0.9781/0.009	4.59/2.56	0.8908/0.094	15.98/11.26
*RFC* − *Net*(All Modules)	**0.9787/0.009**	**3.96/1.79**	**0.9058/0.118**	**15.40/11.19**

#### Research on ablation of multi-scale input and multiple output modules

Our multi-scale input joint FCN takes advantage of the correlation between OD and OC and achieves better performance than the basic FCN. In Table 8, our ‘Backbone’ with multi-scale input and multiple output modules(‘Backbone’+Input+Output) achieves a higher F1 score than the single-scale network ‘Backbone’. In contrast, on the OD, the F1 score increased by 1.03%, and the BLE decreased by 0.38. On the OC, the F1 score increased by 2%, and the BLE decreased by 1.98. It shows that multi-scale input and multiple output modules are useful for guiding early layer training.

#### Ablation research of Polar transformation module

The proposed polar transformation module is used to improve the segmentation performance of the OD and OC. As a contribution of our work, the polar transformation increases the proportion of the OC region. By using polar coordinate transformation, not only the space limitation can be obtained, but also the ratio of the OC region can be increased, which further improves the segmentation performance. We conducted a simulation experiment using polar transformation, and compared the ‘Backbone’ method using polar transformation with the ‘Backbone’ method without using polar transformation. It can be seen from Table 8 that on the OD, the F1 score of the ‘Backbone + PT’ increased by 1.87%, and BLE decreased by 1.42. The effect on the OC is more significant, the F1 score of the ‘Backbone + PT’ increased by 2%, and BLE decreased by 2.48. Please note that ‘Backbone’ and PT perform better than ‘Backbone’ without PT. At the same time, we find that the gain of polar transformation is higher than the gain using multi-scale input and multiple output modules. Polar transformation is particularly helpful for OC segmentation.

#### Ablation research of networks of similar complexity

Researchers have shown that complexity is a manifestation of network functions, and an increase in complexity usually leads to better performance [35]. Therefore, there is a concern that improvements may come from increased network complexity. To alleviate this concern, we compared networks of similar complexity: M-Net [21] and CE-Net [23]. Table 8 shows that our RFC-Net is better. Compared with M-Net [21], the F1 score of the OD increased from 0.9621 to 0.9787, the BLE decreased from 6.07 to 3.96. The F1 score of the OC increased from 0.8513 to 0.9058, and BLE decreased from 17.96 to 15.40. Compared with CE-Net [23], the F1 score of the OD increased from 0.9688 to 0.9787, BLE decreased from 5.04 to 3.96. The F1 score of the OC increased from 0.8699 to 0.9058, and BLE decreased from 16.06 to 15.40.

### 5.2 Can technology work in basic U-Net?

We apply the polar transformation module and the recurrent block to the basic U-Net [6] respectively. Two results of U-Net are reported, one is the basic U-Net [6] used to segment OD and OC, and the other is that U-Net uses our polar transformation module and recurrent block (U-Net+PT+Stack Recurrent Units) to jointly segment OD and OC. As shown in Table 8, compared with the two results, the U-Net with the polar transformation module and the recurrent block achieved better performance. On the OD, the F1 score increased by 1.47%, and the BLE decreased by 0.38. On the OC, the F1 score increased by 2.65%, and the BLE decreased by 3.55. This shows that our proposed technology can work in the basic U-Net.

### 5.3 Limitations and prospects

First of all, in this study, we tried to further expand the OC region through the polar transformation method, and achieved certain results, effectively alleviating the difficulty of determining the OC region in the current method. In the future, the determination methods of OD and OC should be further improved and tested in a larger database.

Secondly, we only analyze the fundus image, which cannot explain the effectiveness of our method in the field of image segmentation, and it is difficult to design representative functions for different applications. So naturally a question is raised: Can the proposed methods and techniques be generalized to other tasks? We conducted ablation experiments carefully and observed positive results. We leave a detailed discussion for future work.

## 6 Conclusions

In this paper, by using the combination of fully convolution network and recurrent convolution network, RFC-Net algorithm is proposed for the OD and OC segmentation. In RFC-Net, a recurrent fully convolution network is applied as the infrastructure. The recurrent unit helps to train the deep architecture, which allows us to design a better FCN network with the same number of network parameters. And downsampling the image naturally constructs a multi-scale input in the encoder path, the multiple output layer is treated as a classifier, generating a segmentation map corresponding to the multi-scale input image. In order to ensure the validity of the output, a multiple output cross entropy loss function is proposed, which can deal with the data imbalance problem in the segmentation image. And the polar transformation effectively improves the segmentation result. The experiment results show that the proposed RFC-Net outperforms some state-of-art algorithm for OD and OC segmentation, such as BCRF, RACE-net, Stack-U-Net, DenseNet FCN and pOSAL.

## Supporting information

S1 File (ZIP)
