# Supplementary figures and images for: Joint disc and cup segmentation based on recurrent fully convolutional network
Source: PLoS One. 2020 Sep 21;15(9):e0238983. doi: 10.1371/journal.pone.0238983 (PMC7505429; doi:10.1371/journal.pone.0238983)

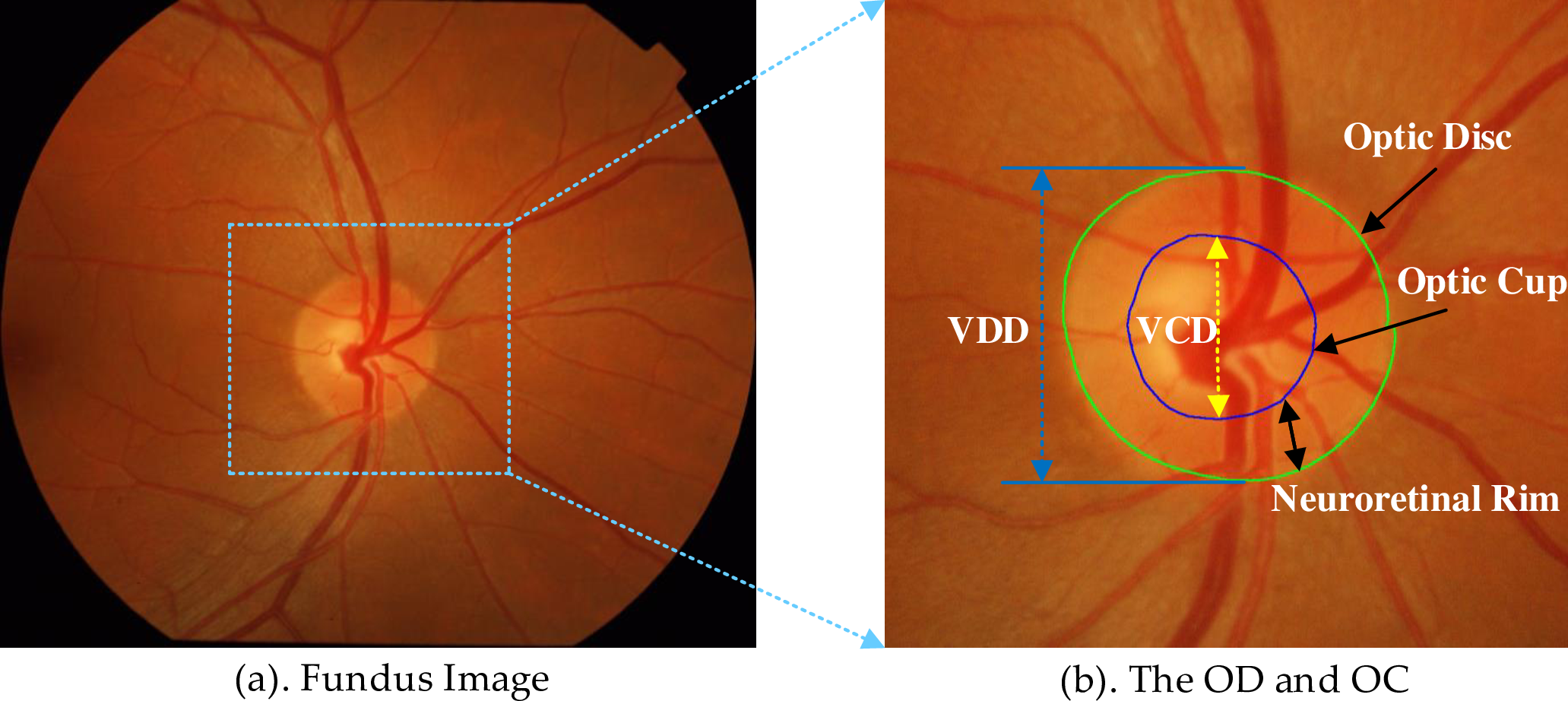

Supplement: S1 File — (ZIP) [file pone.0238983.s001.zip › gaojing-plosone/fig1.tif]

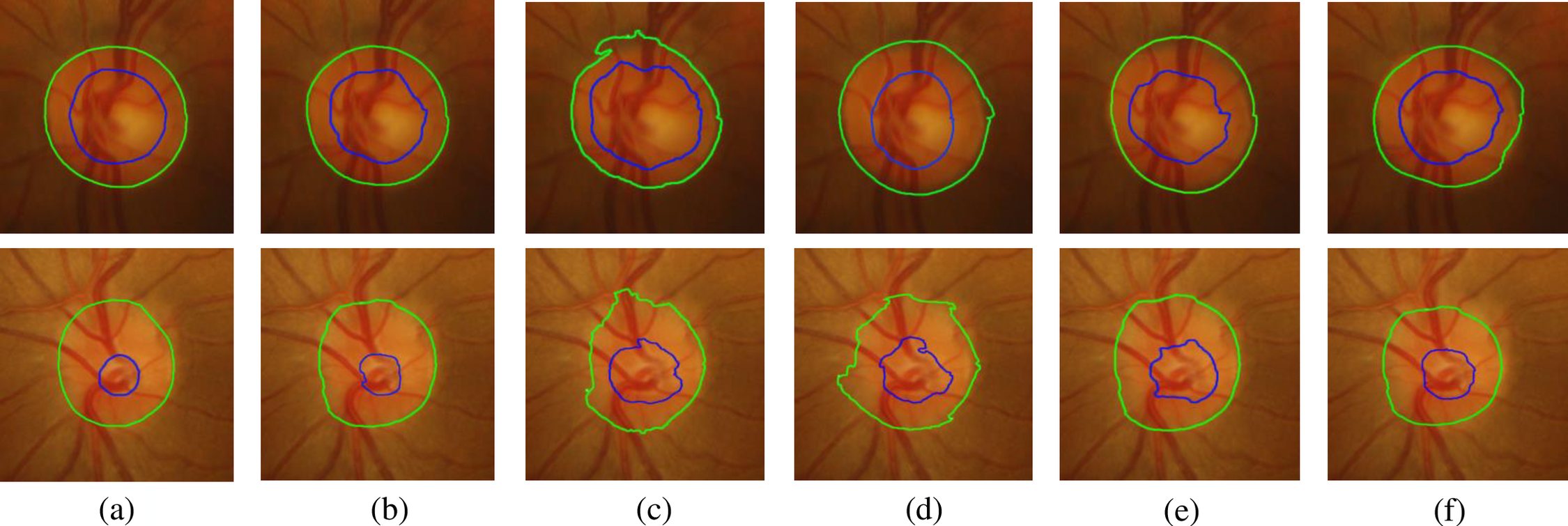

Supplement: S1 File — (ZIP) [file pone.0238983.s001.zip › gaojing-plosone/fig10.tif]

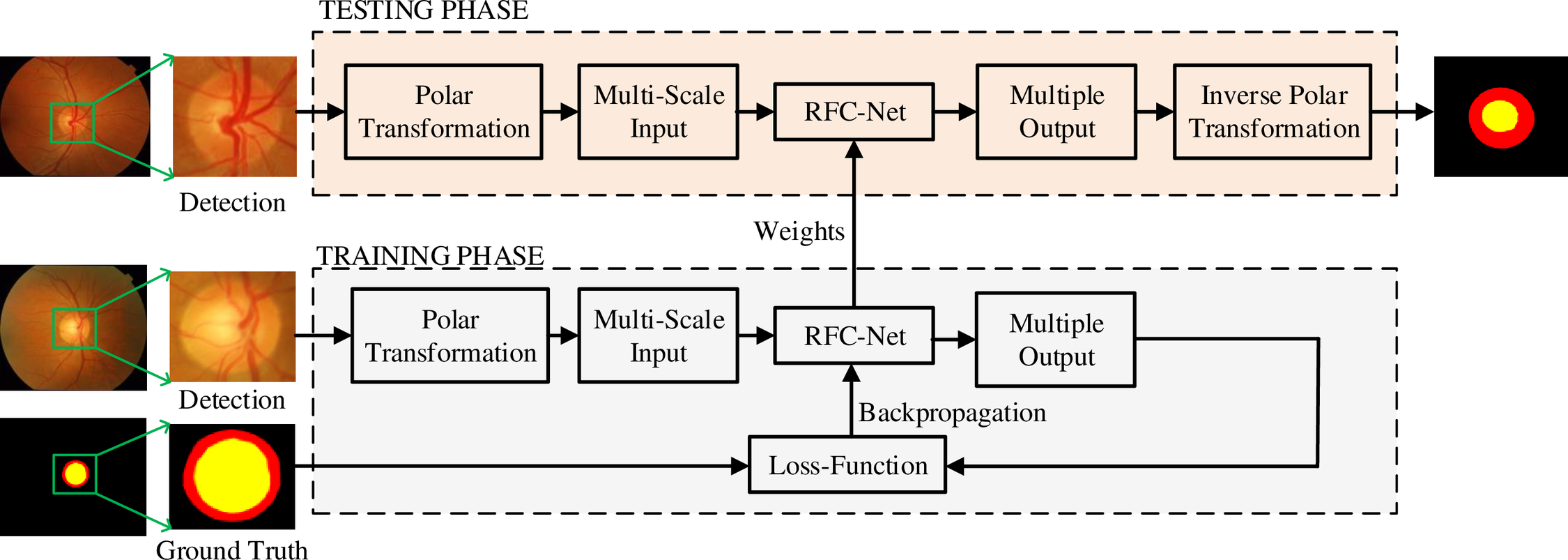

Supplement: S1 File — (ZIP) [file pone.0238983.s001.zip › gaojing-plosone/fig2.tif]

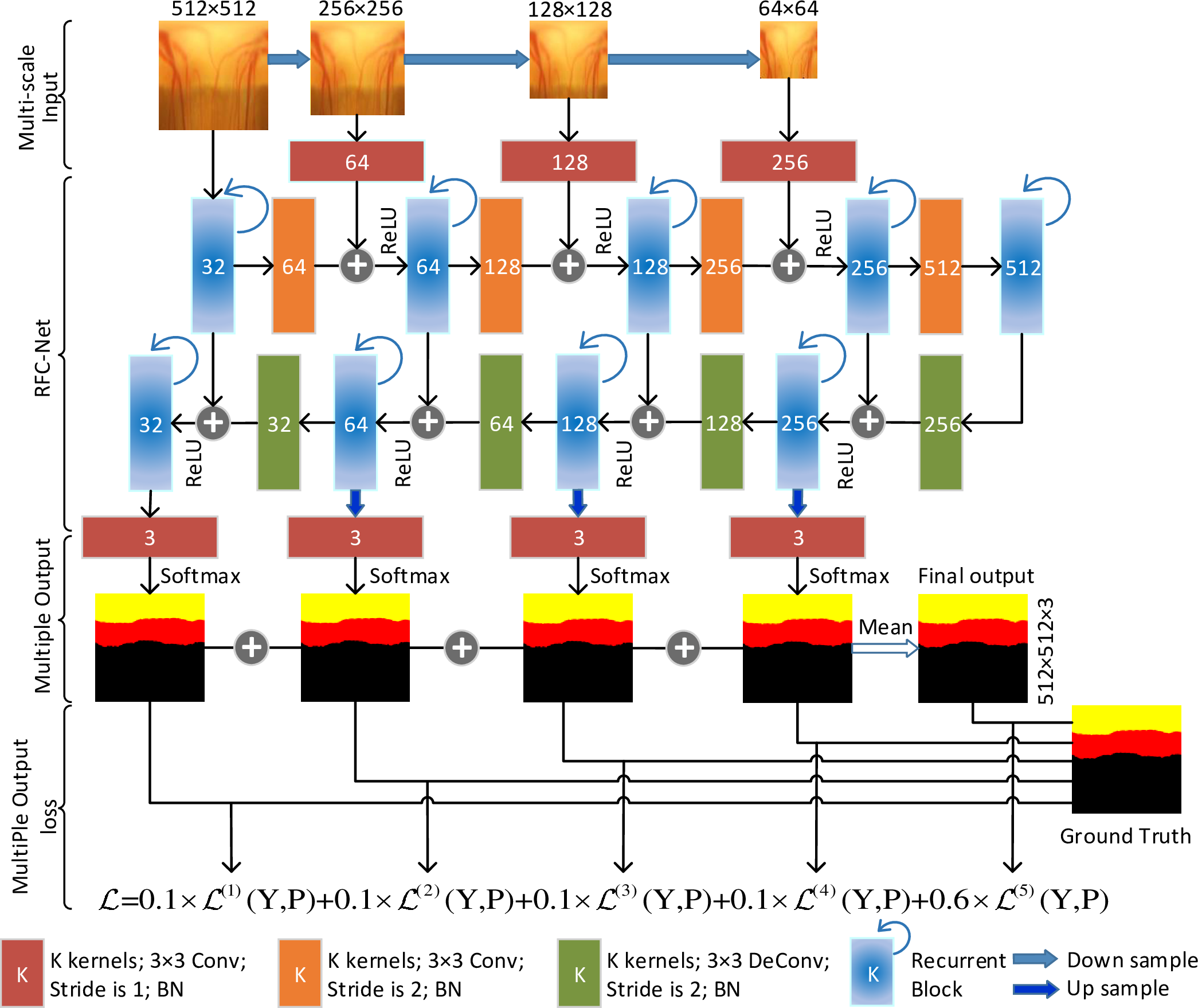

Supplement: S1 File — (ZIP) [file pone.0238983.s001.zip › gaojing-plosone/fig3.tif]

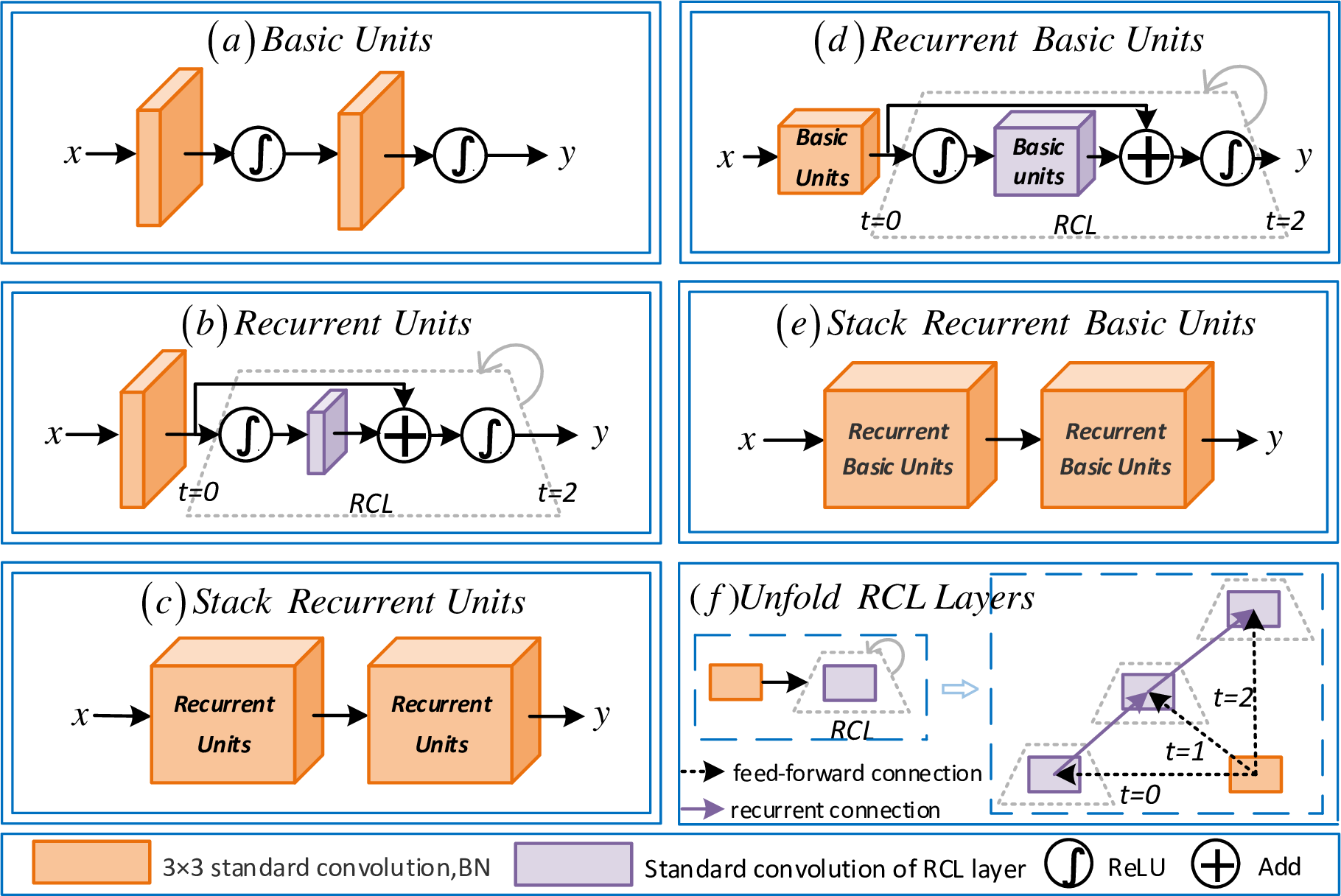

Supplement: S1 File — (ZIP) [file pone.0238983.s001.zip › gaojing-plosone/fig4.tif]

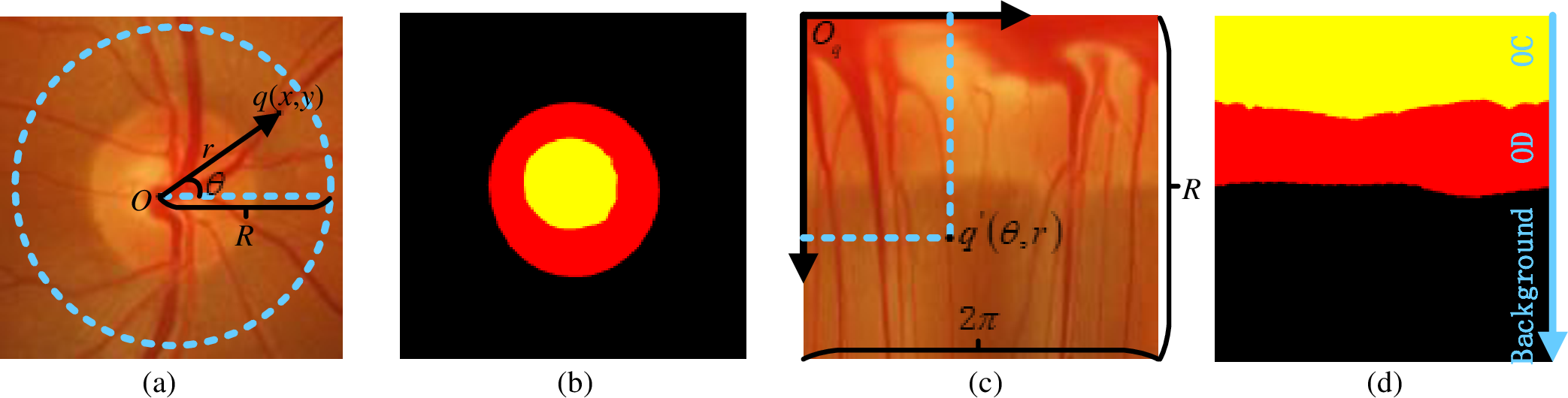

Supplement: S1 File — (ZIP) [file pone.0238983.s001.zip › gaojing-plosone/fig5.tif]

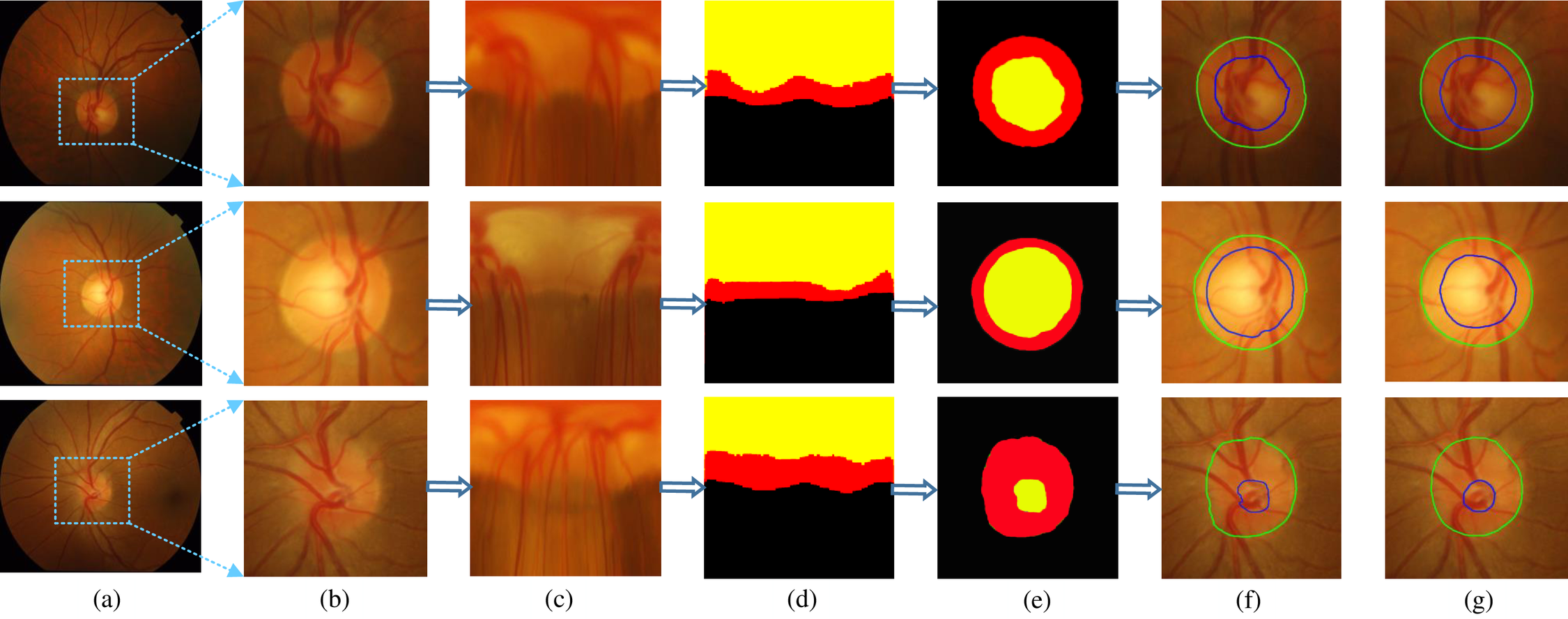

Supplement: S1 File — (ZIP) [file pone.0238983.s001.zip › gaojing-plosone/fig6.tif]

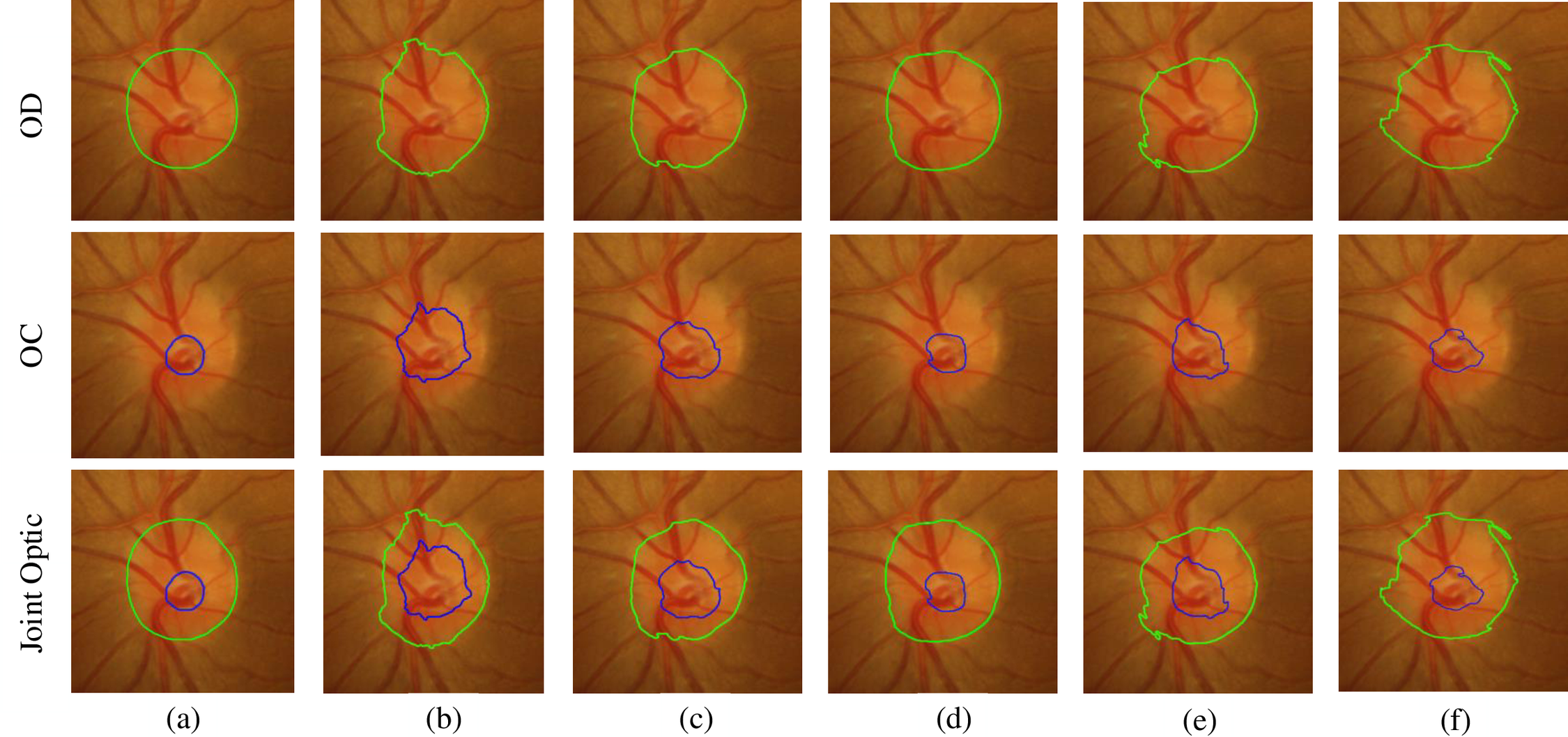

Supplement: S1 File — (ZIP) [file pone.0238983.s001.zip › gaojing-plosone/fig7.tif]

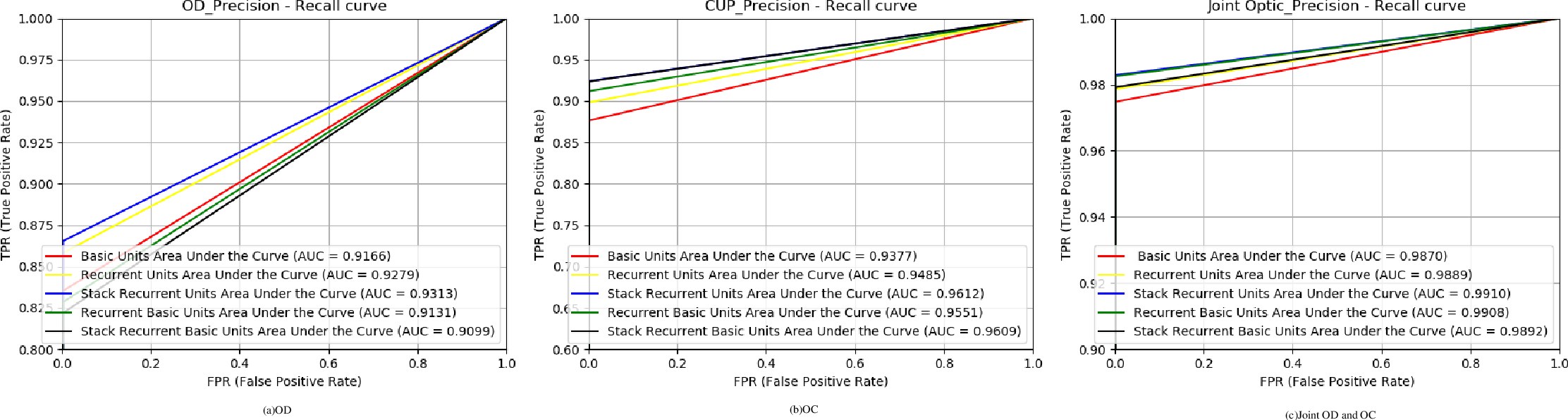

Supplement: S1 File — (ZIP) [file pone.0238983.s001.zip › gaojing-plosone/fig8.tif]

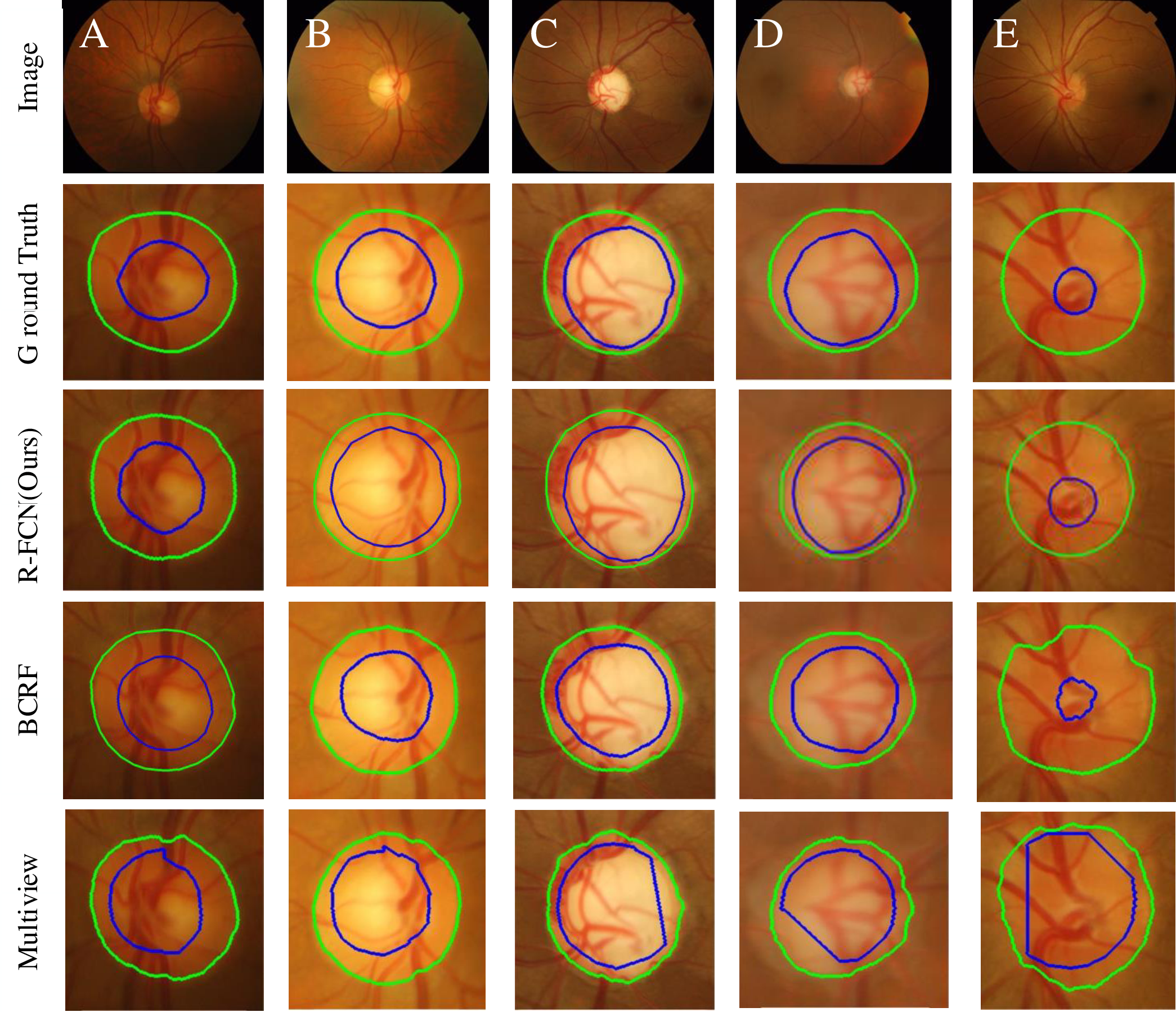

Supplement: S1 File — (ZIP) [file pone.0238983.s001.zip › gaojing-plosone/fig9.tif]
